# Hypervirulent *Klebsiella pneumoniae* employs genomic island encoded toxins against bacterial competitors in the gut

**DOI:** 10.1093/ismejo/wrae054

**Published:** 2024-03-28

**Authors:** Yi Han Tan, Patricio Arros, Camilo Berríos-Pastén, Indrik Wijaya, Wilson H W Chu, Yahua Chen, Guoxiang Cheam, Ahmad Nazri Mohamed Naim, Andrés E Marcoleta, Aarthi Ravikrishnan, Niranjan Nagarajan, Rosalba Lagos, Yunn-Hwen Gan

**Affiliations:** Infectious Diseases Translational Research Programme, Yong Loo Lin School of Medicine, National University of Singapore, 5 Science Drive 2, MD4, Level 2, Singapore 117545, Republic of Singapore; Department of Biochemistry, Yong Loo Lin School of Medicine, National University of Singapore, MD7, 8 Medical Drive, Singapore 117596, Republic of Singapore; Genome Institute of Singapore (GIS), Agency for Science, Technology and Research (A^*^STAR), Singapore 138672, Republic of Singapore; Grupo de Microbiología Integrativa, Laboratorio de Biología Estructural y Molecular BEM, Facultad de Ciencias, Departamento de Biología, Universidad de Chile, Las Palmeras 3425 Ñuñoa, Santiago, Chile; Grupo de Microbiología Integrativa, Laboratorio de Biología Estructural y Molecular BEM, Facultad de Ciencias, Departamento de Biología, Universidad de Chile, Las Palmeras 3425 Ñuñoa, Santiago, Chile; Genome Institute of Singapore (GIS), Agency for Science, Technology and Research (A^*^STAR), Singapore 138672, Republic of Singapore; National Public Health Laboratory, National Centre for Infectious Diseases, 16 Jln Tan Tock Seng, Singapore 308442, Republic of Singapore; Infectious Diseases Translational Research Programme, Yong Loo Lin School of Medicine, National University of Singapore, 5 Science Drive 2, MD4, Level 2, Singapore 117545, Republic of Singapore; Department of Biochemistry, Yong Loo Lin School of Medicine, National University of Singapore, MD7, 8 Medical Drive, Singapore 117596, Republic of Singapore; Infectious Diseases Translational Research Programme, Yong Loo Lin School of Medicine, National University of Singapore, 5 Science Drive 2, MD4, Level 2, Singapore 117545, Republic of Singapore; Department of Biochemistry, Yong Loo Lin School of Medicine, National University of Singapore, MD7, 8 Medical Drive, Singapore 117596, Republic of Singapore; Genome Institute of Singapore (GIS), Agency for Science, Technology and Research (A^*^STAR), Singapore 138672, Republic of Singapore; Grupo de Microbiología Integrativa, Laboratorio de Biología Estructural y Molecular BEM, Facultad de Ciencias, Departamento de Biología, Universidad de Chile, Las Palmeras 3425 Ñuñoa, Santiago, Chile; Genome Institute of Singapore (GIS), Agency for Science, Technology and Research (A^*^STAR), Singapore 138672, Republic of Singapore; Infectious Diseases Translational Research Programme, Yong Loo Lin School of Medicine, National University of Singapore, 5 Science Drive 2, MD4, Level 2, Singapore 117545, Republic of Singapore; Genome Institute of Singapore (GIS), Agency for Science, Technology and Research (A^*^STAR), Singapore 138672, Republic of Singapore; Grupo de Microbiología Integrativa, Laboratorio de Biología Estructural y Molecular BEM, Facultad de Ciencias, Departamento de Biología, Universidad de Chile, Las Palmeras 3425 Ñuñoa, Santiago, Chile; Infectious Diseases Translational Research Programme, Yong Loo Lin School of Medicine, National University of Singapore, 5 Science Drive 2, MD4, Level 2, Singapore 117545, Republic of Singapore; Department of Biochemistry, Yong Loo Lin School of Medicine, National University of Singapore, MD7, 8 Medical Drive, Singapore 117596, Republic of Singapore

**Keywords:** hypervirulent, Klebsiella pneumoniae, colibactin, microcin, gut, commensal, colonization, genomic island

## Abstract

The hypervirulent lineages of *Klebsiella pneumoniae* (HvKp) cause invasive infections such as *Klebsiella*-liver abscess. Invasive infection often occurs after initial colonization of the host gastrointestinal tract by HvKp. Over 80% of HvKp isolates belong to the clonal group 23 sublineage I that has acquired genomic islands (GIs) GIE492 and ICEKp10. Our analysis of 12 361 *K. pneumoniae* genomes revealed that GIs GIE492 and ICEKp10 are co-associated with the CG23-I and CG10118 HvKp lineages. GIE492 and ICEKp10 enable HvKp to make a functional bacteriocin microcin E492 (mccE492) and the genotoxin colibactin, respectively. We discovered that GIE492 and ICEKp10 play cooperative roles and enhance gastrointestinal colonization by HvKp. Colibactin is the primary driver of this effect, modifying gut microbiome diversity. Our *in vitro* assays demonstrate that colibactin and mccE492 kill or inhibit a range of Gram-negative *Klebsiella* species and *Escherichia coli* strains, including Gram-positive bacteria, sometimes cooperatively. Moreover, mccE492 and colibactin kill human anaerobic gut commensals that are similar to the taxa found altered by colibactin in the mouse intestines. Our findings suggest that GIs GIE492 and ICEKp10 enable HvKp to kill several commensal bacterial taxa during interspecies interactions in the gut. Thus, acquisition of GIE492 and ICEKp10 could enable better carriage in host populations and explain the dominance of the CG23-I HvKp lineage.

## Graphical Abstract

**Graphical Abstract:**
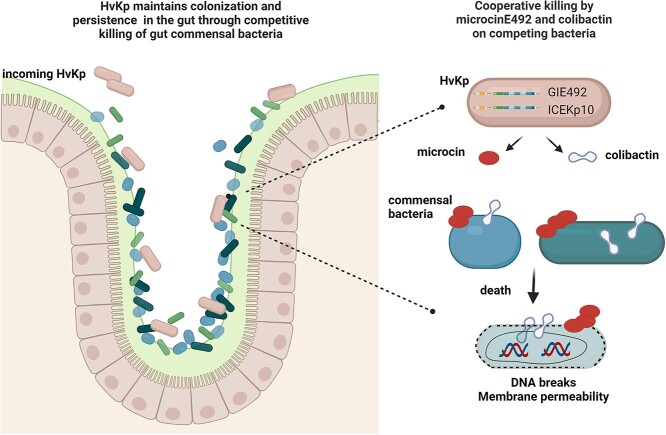

## Introduction

*Klebsiella pneumoniae* is a Gram-negative bacterium characterized as a nosocomial pathogen associated with pneumonia [1]. In contrast, hypervirulent *K. pneumoniae* (HvKp) is community acquired and the major cause of monomicrobial liver abscess in Asia [2, 3]. The dominant lineage of HvKp is clonal group 23-I (CG23-I), comprising mostly sequence type 23 (ST23) strains which cause ~80% of all *Klebsiella*-liver abscess (KLA) infections [4, 5]. Gastrointestinal carriage of HvKp can be high in Asian countries, ranging from 3% to 8% of patients with diarrhea in a Singaporean study to 21.1% of healthy individuals in a Korean study [6, 7]. ST23 HvKp has only been isolated from patients rather than environmental samples [6-9], and gut colonization is strongly associated with the development of KLA [10]. Therefore, factors favoring persistent gastrointestinal carriage of HvKp could benefit its dominance and spread.

Genomic islands (GIs) are horizontally acquired DNA elements integrated in bacterial chromosomes [11]. GIs contribute to the accessory genome of a bacterial species and can contain diverse genetic cargo such as antimicrobial resistance cassettes, metabolic operons, and virulence determinants [11-15]. Most *K. pneumoniae* chromosomes have at least four GIs and some strains possess up to 10 GIs, many encoding known or putative virulence factors [16]. Because GIs can confer beneficial phenotypes, they drive rapid evolution and the success of certain bacterial lineages within a species. Most HvKp strains possess a large virulence plasmid that encodes virulence factors [3, 17-19], as well as several GIs [16]. The GI E492 (GIE492) and integrative conjugative element Kp10 (ICEKp10) are tightly associated with the CG23-I lineage [4, 5, 16]. Currently there is a lack of studies addressing the evolutionary history and prevalence of GIE492 in the *K. pneumoniae* population. Moreover, the contribution of GIE492 and ICEKp10 to the pathogenesis of HvKp in the human host is poorly understood.

GIE492 contains the *mce* gene locus required for the synthesis and export of the bacteriocin microcin E492 (mccE492) [20]. MccE492 is a siderophore-microcin that kills other bacteria via a Trojan-horse mechanism [21]. MccE492 is a siderophore-microcin that enters the periplasm of susceptible prey via catechol siderophore receptors on the outer membrane [22]. Subsequently, mccE492 inserts into the inner membrane to cause cell death [21, 23, 24]. ICEKp10 carries the *ybt* locus encoding yersiniabactin siderophore and the *clb* locus enables the production of colibactin [25-27]. Colibactin is a small molecule alkylating genotoxin that causes double-stranded DNA breaks (DSBs). Gut carriage of *clb*-positive bacteria is associated with colorectal cancer [25, 28, 29]. Recent studies have shown that beyond its effect on the host, colibactin-producing *Escherichia coli* can utilize colibactin to kill other bacteria [30-32].

We profiled the evolution and prevalence of GIE492 in *K. pneumoniae* and found eight main variants of GIE492, of which a specific variant is co-associated with ICEKp10 in the hypervirulent CG23-I *K. pneumoniae* lineage. Furthermore, we discovered that GIE492 and ICEKp10 play cooperative roles during gastrointestinal colonization of SGH10, a representative clinical isolate of CG23-I [4]. Colibactin causes changes to the gut microbiome that benefit HvKp colonization. Our results support a model where GIE492 and ICEKp10 enable HvKp to compete effectively with other bacterial species in the ecological niche of the gut. Thus, we postulate that the acquisition of GIE492 and ICEKp10 is likely a contributing factor to the emergence ofje CG23-I as the dominant lineage of HvKp.

## Materials and methods

### Bacterial cultures

All bacterial strains used in this study are listed in Supplementary Table 1. Facultative anaerobes were revived from glycerol stocks on Lysogeny broth agar (ThermoFisher Scientific) and grown at 37°C under oxic conditions. Anaerobic bacteria were grown in Reinforced Clostridial Medium broth and agar (Sigma-Aldrich) under anoxic conditions at 37°C. Facultative anaerobes were grown at 37°C with shaking in Dulbecco’s modified Eagle’s medium (Life Technologies) with 10% fetal bovine serum (Singlab) unless otherwise specified.

Where necessary, 50 μg/ml kanamycin sulfate (Sigma-Aldrich), 10 μg/ml chloramphenicol (Sigma-Aldrich), 20 μg/ml tetracycline (Sigma-Aldrich), 10 μg/ml trimethoprim (Sigma-Aldrich), 10 μg/ml gentamicin sulfate (Sigma-Aldrich), and 50 μg/ml carbenicillin sodium salt (Sigma-Aldrich) were used in media.

### Mouse experiments

Female C57BL/6 mice aged 7–8 weeks from InVivos were inoculated with 10^5^ CFU of *K. pneumoniae* in 1 × Phosphate-buffered saline (PBS) via intraperitoneal injection to establish systemic infection. Mice were sacrificed at 30 hours post infection (hpi), and organ bacterial loads were enumerated by plating appropriate dilutions of organ homogenates on *Klebsiella* selective agar (KSA) supplemented with 50 μg/ml carbenicillin.

To establish gastrointestinal colonization, mice were gavaged with 2.5 mg of ampicillin (Sigma-Aldrich) in 100 μl 1 × PBS daily for 5 days. On the subsequent day, mice were infected with 5 × 10^6^ colony forming units (CFU) of *K. pneumoniae* in 100 μl 1 × PBS via oral gavage. Fresh stools were collected from each mouse, and bacterial load was enumerated by plating on KSA.

To establish gut translocation, mice were gavaged with a cocktail of 2.5 mg ampicillin sodium salt, 0.2 mg of vancomycin hydrochloride, 1 mg of metronidazole, and 0.16 mg of colistin sulfate in 400 μl of 1 × PBS daily for 5 days. On the following day, mice were switched from normal drinking water to 15% Miralax (Bayer) in Milli-Q water. Mice were then orally gavaged with 10^9^ CFU of *K. pneumoniae* 1 day later. Mice were weighed and scored for sickness daily. Mice reaching termination criteria or 20% weight loss were euthanized. Procedures were approved by the Institutional Animal Care and Use Committee at National University of Singapore (R18–0252).

### Stool DNA extraction and Illumina sequencing of metagenomic libraries

DNA was extracted from frozen mouse stools using the QIAamp PowerFecal Pro DNA Kit (Qiagen). The purified DNA underwent NGS library construction steps using NEBNext Ultra II FS DNA Library Prep Kit (New England BioLabs) according to the manufacturer’s instructions. Post fragmentation, adaptor-ligation was performed using Illumina-compatible adaptors, diluted 10-fold as per kit’s recommendations prior to use. Postligation, samples were purified using Agencourt Ampure XP beads (Beckman Coulter) in a 7:10 beads-to-sample volume. Unique barcode indexes were added to each purified sample and amplified for 12 cycles under recommended kit conditions to achieve multiplexing within a batch of samples. Finally, each library sample was assessed for quality based on fragment size and concentration using the Agilent D1000 ScreenTape system and adjusted to identical concentrations by means of dilution and volume-adjusted pooling. The multiplexed sample pool was paired-end (2 × 151 bp) sequenced on the HiSeq X Ten system (Illumina). All sequencing was done in the Novogene sequencing facility at the Genome Institute of Singapore in accordance with standard Illumina sequencing protocols. An average of 14 893 204 raw reads, and an average of 7 446 602 paired reads were generated for each sample.

### Statistical analysis

Data were plotted using Graphpad Prism 9.0 (La Jolla California USA, www.graphpad.com). Long read-sequencing data were plotted using R (v4.1.2; R Core Team 2021). Dunnett’s multiple comparison’s tests were used to compare means and standard deviations (SDs) in *in vitro* assays. For *in vivo* experiments, Dunnett’s multiple comparison’s test was used to compare geometric means relative to SGH10. A *P* value <.05 was considered statistically significant and denoted as ^*^, and a *P* value <.01 is denoted as ^**^. The Mantel–Cox test was performed to determine if there were significant differences in mortality between the groups. Microbiome analysis methods and *K. pneumoniae* phylogenomic analyses are described in Supplemental Methods. Bacterial mutant generation and bacterial prey-predator cocultures are also described in Supplemental Methods.

## Results

### Evolutionary history of GIE492 and its association with ICEKp10 in *K. pneumoniae* CG23

GIE492 was first characterized in the clonal group 35 (CG35) isolate *K. pneumoniae* RYC492 [20, 21, 33]. However, little is known about this GI in other lineages of *K. pneumoniae* and its potential roles in pathogenesis. We investigated the structure, distribution, and evolutionary history of GIE492 in *K. pneumoniae* and its relationship with CG23-I.

**Figure 1:**
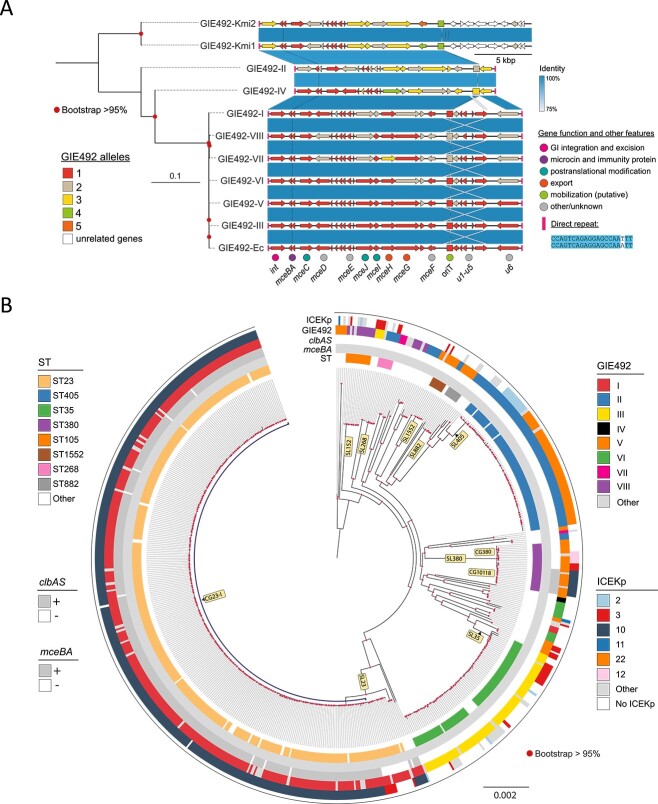
Genomic structure and phylogenetic distribution of GIE492 in *K. pneumoniae*; (A) sequence alignment of GIE492 structural variants of *K. pneumoniae* (GIE492-I to GIE492-VIII), as well as GIE492 variants in *K. michiganesis* (GIE492-Kmi1 and 2) and *E. coli* (GIE492-Ec); (B) distribution of GIE492 and ICEKp variants in 588 GIE492+ *K. pneumoniae* genomes; a maximum-likelihood tree of the GIE492+ genomes was plotted based on core genome multilocus sequence alignment (cgMSA) of the scgMLSTv2 scheme; colors indicating GIE492-I and GIE492-II indicate their respective one-locus variants; ICEKp10 and ICEKp12 colored slots also include versions of these elements predicted as incomplete by Kleborate.

We produced a reference sequence for GIE492 by assembling the complete genome of *K. pneumoniae* RYC492 with Illumina and nanopore sequencing. Using this reference, we searched for GIE492 in 12 433 KpSC genomes from (1) the RefSeq database, (2) a multi-centre clinical trial studying KLA patients in Singapore (A-KLASS) [34], (3) a set of bloodstream infection isolates from Southeast Asia [35], (4) the CG23 genome set analyzed by Lam *et al*. [4], and (5) the Murray collection of bacterial isolates from the preantibiotic era. We identified 657 GIE492+ genomes in *K. pneumoniae sensu stricto* species (Supplementary Table 2) and discovered eight main GIE492 variants based on sequence comparison and allele calling for the 23 genes in GIE492 [20]. Six variants were similar in length to GIE492-III (first characterized in RYC492), and GIE492-II and GIE492-IV lacked a ~4.6-kbp internal region comprising the u1–u5 genes of unknown function (Fig. 1A). The remaining variants included up to four main variable alleles for some genes (Supplementary Data 1). The eight GIE492 variants were flanked by 100% conserved 20-bp direct repeats, carried the P4-like integrase-coding gene, and had the putative transfer origin (oriT). Other less frequent variants included insertion sequences (mainly ISKpn72 and ISKpn74), likely leading to variable deletions (Supplementary Fig. 1).

We also searched for GIE492 in a set of 82 206 *Enterobacterales* genomes from the RefSeq database. Other than in *K. pneumoniae*, GIE492 was only found in two *E. coli* (GIE492-Ec) and two *Klebsiella michiganensis* genomes (GIE492-Kmi1 and 2) (Fig. 1A). The latter carried a divergent GIE492 with more similarity to GIE492-II, with rearrangements and different gene content in the 3′ region, lacking the direct repeat (Fig. 1A). Thus, GIE492 is highly restricted to *K. pneumoniae*, although it may be transferred to other related species with relatively low frequency.

To investigate the phylogenetic relationships among the GIE492+ strains, sublineages (SLs; threshold: 190 allelic mismatches) and clonal groups (CGs; threshold: 43) were identified using cgMLST analysis. We also determined sequence type (ST) according to the classical seven-gene MLST scheme for better comparison with previous phylogroup definitions. SL23, SL405, SL35 (including RYC492), SL380, and SL152 are most prevalent, and are associated mainly with variants I, II, III, V, and VIII, respectively (Fig. 1B). This distribution suggests an early acquisition of this GI in different *K. pneumoniae* lineages, followed by vertical transmission within each lineage. The presence of the shortened variant GIE492-II in seven relatively distant lineages suggests an early occurrence of the putative deletion event leading to the shorter GIE492 variants (Fig. 1B, Supplementary Table 2). The oldest strains bearing GIE492 (found in the Murray collection), ERR230425 (1932, CG23), and GCA_022308495.1 (1940, CG35) possessed Variants I and III, respectively, further supporting the hypothesis that longer GIE492 variants are ancestral.

In terms of the co-occurrence of GIE492 and ICE*Kp* variants in our dataset, a high proportion (82%) of GIE492+ genomes carried an ICE*Kp* element (Supplementary Table 2). GIE492-I and ICEKp10 co-occur in the globally disseminated HvKp subclade CG23-I, corroborating earlier observations (Fig. 1B) [4]. CG23-I (described initially based on SNPs) mainly included isolates with LIN codes 0_0_429_0_4/5/6/8/9/44, and is also highly associated with the alleles *ybt1*, *clb2*, and the virulence plasmid KpVP-1. Moreover, GIE492-V and ICE*Kp*10 also co-occurred in CG10118 (SL380), although few SL380 genomes were present in our dataset (Fig. 1B). Both SL23 and SL380 lineages are associated with KLA, although SL380 is a minor HvKp lineage [36, 37]. In all GIE492 + ICEKp10+ isolates, the colibactin and microcin immunity genes *clbS* and *mceB* were present (Supplementary Table 2). We did not find GIE492-ICEKp10-genomes that carried *clbS* or *mceB* (Supplementary Table 2).

GIs are unstable elements that may be easily lost after acquisition, but positive selection maintains GIs in bacterial lineages if their genetic cargo confers benefits. The co-occurrence of specific GIE492 variants and ICEKp10 in two hypervirulent lineages of *K. pneumoniae* suggests that two independent acquisitions of GIE492 occurred and were preserved in these hypervirulent clades. CG23-I is the dominant lineage of HvKp, and GIE492 and ICEKp10 may have contributed to its success.

### SGH10 produces functional mccE492 and colibactin

**Figure 2:**
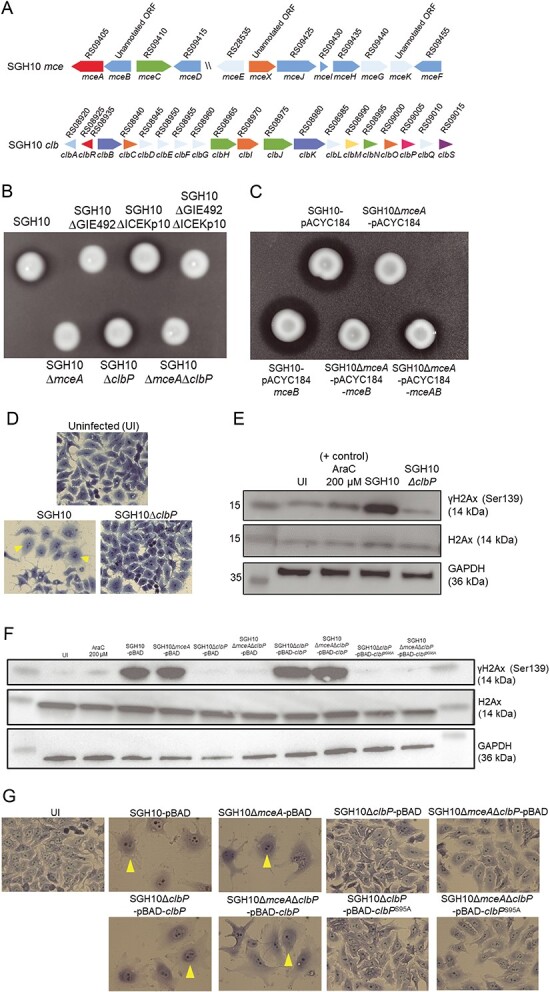
The clonal group-23 HvKp strain SGH10 produces active microcin E492 and colibactin; (A) genetic organization of the *mce* and *clb* loci in SGH10; *mceB* is an unannotated open reading frame (ORF) from base pairs 1 924 637–1 924 924, *mceX* is an unannotated ORF from base pairs 1 930 032–1 930 139, and *mceK* is an unannotated ORF from base pairs 1 935 889–1 936 298; (B) an agar diffusion assay was used to confirm if SGH10 produced a functional mccE492; SGH10 and mutant strains were spotted on a lawn of *E. coli* MG1655 prey; a growth inhibition halo can be observed around microcin producing strains; (C) the agar diffusion assay was used to demonstrate complementation of SGH10Δ*mceA*; SGH10 and SGH10Δ*mceA* containing a control plasmid pACYC184, pACYC184-*mceB*, or pACYC184-*mceAB* were spotted on a lawn of prey *E. coli* MG1655; strains producing mccE492 are surrounded by a halo of killed *E. coli*; (D) light micrographs of methylene blue-stained HepG2 cells infected with SGH10 and SGH10∆*clbP* at an MOI of 50:1 at 48 hpi; the cells were imaged at 20× magnification, and yellow arrows indicate megalocytotic cells with distended nuclei and cell bodies; (E) measurement of γH2Ax in Kp-infected cells; HepG2 cells were infected with the complemented SGH10 and mutant strains at an MOI of 50:1, and cytarabine (AraC) was used as a positive control; cell lysates were harvested at 8 hpi for western blotting to quantify γH2Ax (Ser139, phosphorylated at serine 139), and H2Ax and GAPDH were included as loading controls; (F) complementation of SGH10∆*clbP*. HepG2 cells was infected with SGH10, SGH10∆*mceA*, SGH10∆*clbP*, and SGH10∆*mceA*∆*clbP* carrying either the pmLBAD (pBAD) control vector or pBAD expressing either wildtype ClbP or ClbP^S95A^ at an MOI of 50:1; 200 μM cytarabine (AraC) was used as a positive control for DNA damage and UI denotes uninfected cells; cell lysates were harvested at 8 hpi for western blotting to quantify γH2Ax, H2Ax, and GAPDH as a control; (G) at 48 hpi, HepG2 cells infected in the same manner as in (F) were fixed and stained with Giemsa and imaged at 20× magnification; megalocytotic cells are indicated with arrows.

SGH10 possesses GIE492-I and ICEKp10 [4]. The *mce* and *clb* genetic loci in SGH10 encode microcin E492 and colibactin, respectively (Fig. 2A). We created markerless deletion mutants of GIE492, ICEKp10, *mceA*, and *clbP*. Using an agar diffusion assay, we demonstrated that SGH10 makes a functional mccE492 that inhibits the growth of *E. coli* prey (Fig. 2B)*.* The deletion mutants in GIE492 and *mceA* (microcin E492 precursor) did not kill *E. coli* (Fig. 2B). Because expressing excess *mceA* alone results in self-intoxication, we complemented SGH10Δ*mceA* by expressing both the microcin precursor and immunity protein *(mceAB*) under the control of the native promoter (Fig. 2C). The complemented strain killed *E. coli* prey, whereas SGH10Δ*mceA* expressing *mceB* alone or an empty vector did not (Fig. 2C).

To validate SGH10’s ability to make colibactin, HepG2 cells were infected with SGH10. Infected cells exhibited distended nuclei and cell bodies, characteristic of the megalocytotic phenotype induced by the genotoxic effect of colibactin (Fig. 2D) [38]. Conversely, cells infected with ∆*clbP*, where the enzyme responsible for maturation of colibactin has been deleted appeared similar in morphology to uninfected cells (Fig. 2D). γH2AX accumulation is a marker of double-stranded DNA breaks in mammalian cells [39]. We observed a strong γH2AX signal in HepG2 cells infected with SGH10 but not in cells infected with SGH10Δ*clbP* (Fig. 2E). Complementation of SGH10Δ*clbP* with wildtype *clbP* restored genotoxicity (Fig. 2F). Expression of ClbP^S95A^, a catalytically inactive ClbP, did not restore γH2AX accumulation or megalocytosis in SGH10Δ*clbP* (Fig. 2F and G). Thus, the genotoxic effect of SGH10 is dependent on the production of mature colibactin.

### MccE492 and colibactin are important for gut colonization

**Figure 3:**
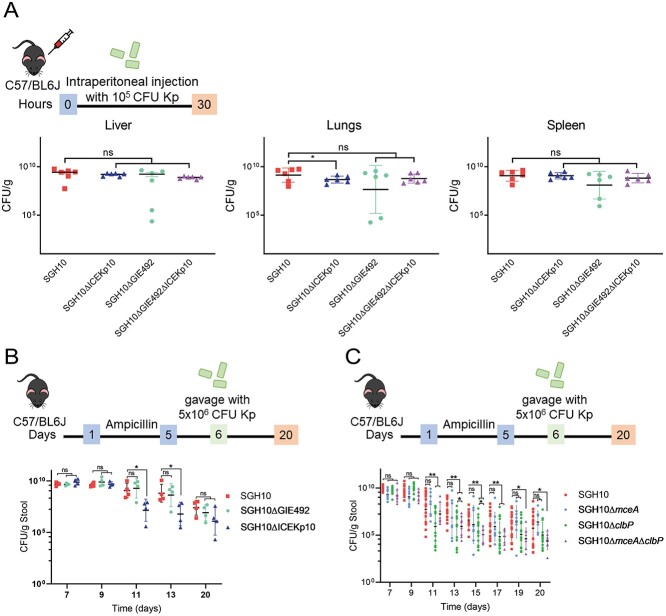
The roles of mccE492 and colibactin during HvKp infection; a systemic infection was established via intraperitoneal injection of C57BL/6 mice with 10^5^ CFU of SGH10, SGH10ΔGIE492, SGH10ΔICEKp10, and SGH10 ΔGIE492ΔICEKp10; bacterial loads were quantified in the liver, lungs, and spleen at 30 hpi; (B) mice were colonized with SGH10, SGH10ΔGIE492, SGH10ΔICEKp10, as well as C SGH10Δ*mceA*, SGH10Δ*clbP* and SGH10Δ*mceA*Δ*clbP*; C57BL/6 mice were treated with ampicillin for 5 days before oral gavage with 5 x 10^6^ CFU SGH10 and mutants; stool bacterial loads were quantified at appropriate intervals; mouse infection experiments were performed *n* = 2–4 times; the geometric mean and SD were plotted, and Dunnett’s multiple comparisons test was performed on log-transformed CFU values to determine differences in means; ^*^ denotes *P* < .05 and ^**^ denotes *P* < .01.

Because GIE492 and ICEKp10 co-associate with the CG23-I lineage, we hypothesized that these GIs are important during disseminated infection. There were no defects in the ability of SGH10ΔGIE492 and SGH10ΔICEKp10 to infect the liver and spleen in a murine model of systemic infection, though there was a modest reduction in SGH10ΔICEKp10 in the lungs (Fig. 3A). These GIs are dispensable during systemic infection at the infectious dosage we used.

We then examined whether GIE492 and ICEKp10 are important during gastrointestinal colonization using a murine model of infection that we previously established [40]. At Days 11 and 13, stool CFU of SGH10ΔICEKp10 was significantly lower than SGH10 (Fig. 3B). There were no differences in the stool CFU of SGH10ΔGIE492 and SGH10 (Fig. 3B). Hence, ICEKp10 but not GIE492 is important for gastrointestinal persistence.

ICEKp10 and GIE492 contain other genetic content besides *mce* and *clb*. We then created ∆*mceA* and ∆*clbP* to determine the specific roles of mccE492 and colibactin. SGH10∆*mceA* colonized as well as SGH10 (Fig. 3C). However, SGH10Δ*clbP* and SGH10Δ*mceA*Δ*clbP* were attenuated in their ability to persist in the gut from Days 11 till 17 (Fig. 3C). Interestingly, stool bacterial loads of SGH10Δ*mceA*Δ*clbP* were significantly lower than SGH10Δ*clbP* on Days 13 and 15 (Fig. 3C). Even on Days 19 and 20, bacterial loads of SGH10Δ*mceA*Δ*clbP* were the lowest among all groups (Fig. 3C). Gut colonization appears to be colibactin-driven with mccE492 playing a supporting role by amplifying the effect of colibactin, as microcin alone did not affect colonization.

**Figure 4:**
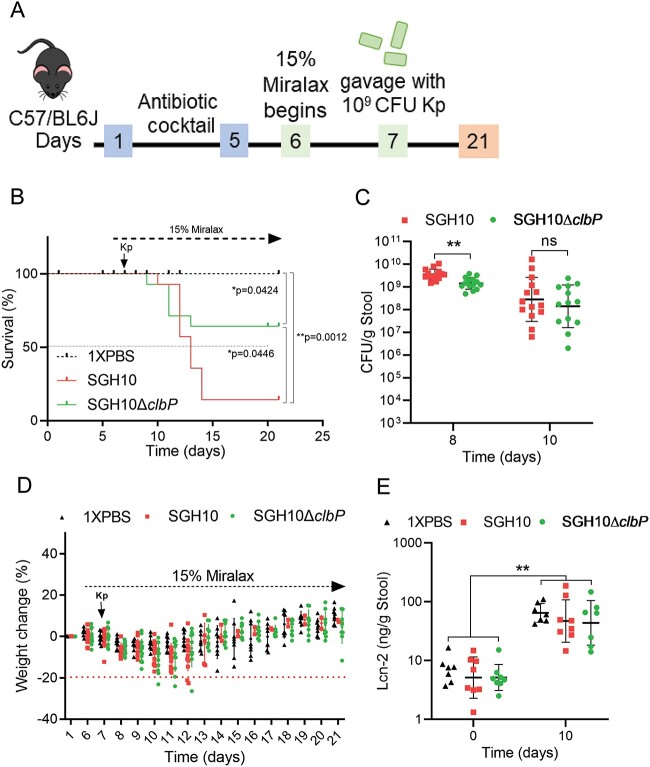
The role of colibactin in bacterial translocation; (A) a murine model of HvKp translocation was used to determine the lethality of SGH10 or SGH10Δ*clbP*; 1 × PBS was used as a control; (B) the mice were scored daily for signs of sickness and culled when they reached termination criteria; a survival curve of mortality induced by SGH10 and SGH10Δ*clbP* was plotted; The Mantel–Cox test was performed to determine if there were significant differences in mortality between the groups; (C) stool bacterial-loads of SGH10 and SGH10Δ*clbP* infected mice; (D) weight change (%) relative to the initial weight of the mice during the experiment was plotted; mice were culled when they met termination criteria or if weight loss was greater than 20%; (E) stool lipocalin-2 was measured by ELISA; the geometric mean and SD are plotted for (C and E), and mouse experiments were performed *n* = 2 times; Dunnett’s multiple comparisons test was performed on log-transformed values to determine differences in means; ^*^ denotes *P* < .05, and ^**^ denotes *P* < .01.

We developed a murine model of *K. pneumoniae* translocation dependent on an antibiotic cocktail and Miralax, a mild osmotic laxative that induces thinning of the gut mucosa [41] (Supplementary Fig. 2A). This treatment establishes high rates of HvKp translocation to the liver, lungs, and spleen (Supplementary Fig. 2B). We then determined whether colibactin is important in the context of gastrointestinal translocation (Fig. 4A). About 85.7% of the mice infected with SGH10 succumbed to infection, whereas SGH10Δ*clbP* killed only 35.7% of infected mice (Fig. 4B). There was a significant difference in stool bacterial loads on Day 8 (Fig. 4C), suggesting that attenuation of SGH10Δ*clbP* in this model may be due to transient differences in gut colonization. It is also possible that colibactin causes damage to the intestinal epithelium and increases translocation of SGH10. However, we observed no significant differences in weight as an indication of sickness, or stool lipocalin-2, a marker of inflammation (Fig. 4D and E).

### Colibactin-driven changes in the gut microbiome

**Figure 5:**
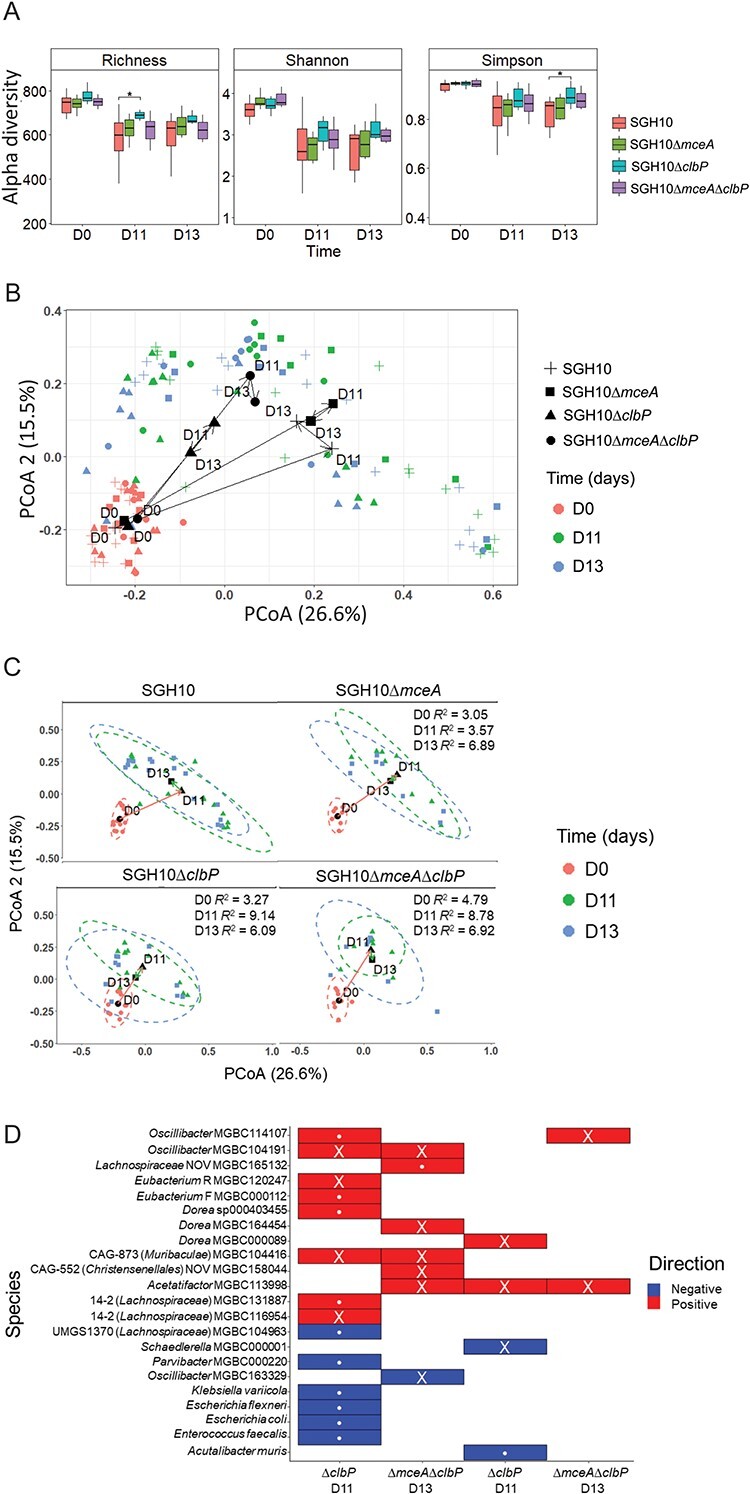
Colibactin-dependent alteration of the murine gut microbiome; (A) differences in α-diversity between mice colonized with SGH10 or mutants; richness, Shannon and Simpson index values were plotted and linear mixed models that adjust for batch were used to determine if there were significant differences between groups; the *P* value of SGH10 vs. SGH10Δ*clbP* at D11 and D13 is .012 and .027 respectively; (B) PCoA (principal Co-ordinates analysis) was performed on Bray–Curtis distance data as a measure of β-diversity, and PCoA1–PCoA2 were plotted for all groups; centroids of each group are indicated with black dots and connected with arrows; (C) PCoA1–PCoA2 plots of Bray–Curtis distance data (β-diversity) were individually plotted by group; ellipses which represent 90% confidence interval are drawn around each timepoint and arrows are plotted connecting the centroids of each timepoint within groups; PERMANOVA analysis of the Bray–Curtis data determined that there were significant differences in β-diversity between SGH10 vs. SGH10Δ*clbP* and SGH10 vs. SGH10Δ*mceA*Δ*clbP* at D11; *R*^2^ and *P* values are listed in Supplementary Table 3; the *R*^2^ value represents the percentage of variance in the data explained by the distinctions between groups when comparing each mutant to the wild type; a higher *R*^2^ indicates a more pronounced impact of the group differences on the dissimilarity observed among samples; the alpha value cutoff was set to 0.05; (D) differential abundance analysis was conducted using MaAsLin2, comparing SGH10 vs. SGH10Δ*clbP* and SGH10 vs SGH10Δ*mceA*Δ*clbP* at D11 and D13; colored cells marked with “•” correspond to FDR-adjusted *P* value <.1; taxa which are positively or negatively abundant relative to SGH10 are plotted, and *P* values are listed in Supplementary Table 4; the *Oscillibacter*, *Lachnospiraceae*, *Eubacterium*, *Dorea*, *Acetatifactor* taxa belong to the *Clostridiales* order, whereas *Muribaculae* belongs to the *Bacteroidales* order and *Parvibacter* belongs to the *Eggerthellales* order. *Klebsiella variicola* and *Escherichia* belong to the *Enterobacterales* order, *E. faecalis* to the *Lactobacillales* order, and *Acutalibacter muris* to the *Eubacteriales*.

We hypothesized that mccE492 and colibactin cause changes in the gut microbiome that benefit *K. pneumoniae* colonization. To determine whether gut bacterial taxa were affected, shotgun metagenomic sequencing was conducted on samples from the experiment in Fig. 3C. Stools were collected before the microbiome was perturbed with ampicillin (Day 0), and when the greatest difference in the abundance of *K. pneumoniae* was observed between groups (Days 11 and 13). We observed a colibactin-associated reduction of α-diversity in the gut microbiome and not with other mutants on Days 11 (Richness) and 13 (Simpson) (Fig. 5A). Subsequently, we plotted β-diversity (Bray–Curtis distance) on a PCoA plot (Fig. 5B). The points at Day 0 cluster more tightly (Fig. 5B), but after ampicillin treatment and colonization with *K. pneumoniae*, there is a shift that differs depending on bacterial mutants (Fig. 5C). Permutational multivariate ANOVA (PERMANOVA) analysis was performed on the Bray–Curtis distances between samples from different groups and indicated that there were significant differences in gut microbiome composition of SGH10 with SGH10∆*clbP* on both Days 11 and 13 (*R*^2^ = 9.14, *P* = .011; *R*^2^ = 6.09, *P* = .026), as well as between SGH10 and SGH10∆*mceA*∆*clbP* infected mice, but only on Day 11 (*R*^2^ = 8.78, *P* = .023) (Fig. 5C). We did not observe significant differences in microbiome composition upon infection with SGH10Δ*mceA* compared to SGH10, and smaller *R*^2^ values were observed for SGH10Δ*mceA* (Fig. 5C), indicating that the primary driver of changes in the gut microbiome is colibactin.

We plotted relative abundance of microbial taxa in the groups at the Family level but did not observe significant differences at this taxonomic rank (Supplementary Fig. 3). Subsequently, MaAsLin2 was used to conduct differential abundance analysis at different time points. In mice colonized with SGH10∆*clbP*, multiple taxa of *Oscillibacter*, *Lachnospiraceae* (14-2), *Christensenella* (CAG-552), and *Muribaculaceae* (CAG-873) were enriched relative to mice colonized with SGH10 (Fig. 5D, Supplementary Fig. 4). Conversely, there was a reduction in the abundance of *Enterobacterales*, *K. pneumoniae*, *Enterococcus faecalis*, and *E. coli* in mice colonized with SGH10∆*clbP* relative to mice colonized with SGH10 (Fig. 5D, Supplementary Fig. 4, Supplementary Table 4). Although PERMANOVA analysis showed that the differences in microbiome composition were insignificant between SGH10 and SGH10∆*mceA*∆*clbP* colonized mice on Day 13 (Fig. 5C), we observed enrichment in one *Oscillibacter* and one *Acetatifactor* taxon relative to wildtype (Fig. 5D). The colibactin-induced changes in gut microbiome composition may be due to colibactin-mediated inhibition of these taxa.

### *K. pneumoniae* kills other bacteria via microcin- and colibactin-dependent mechanisms

**Figure 6:**
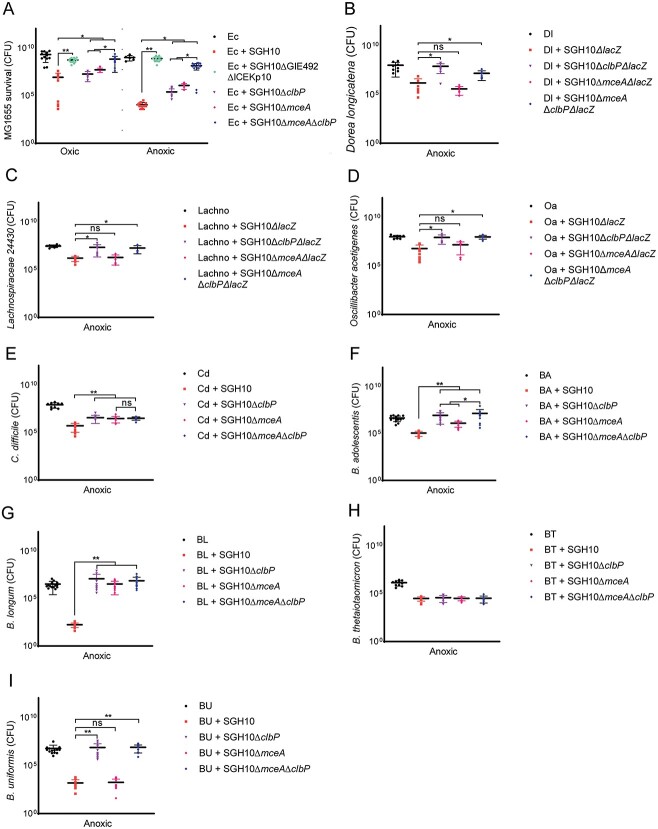
HvKp can utilize mccE492 and colibactin to kill other bacteria; (A) survival of *E. coli* MG1655 (Ec) when competed with *K. pneumoniae* on solid media for 24 h; (B) sensitivity of anaerobic bacteria to killing by mccE492 and colibactin; we competed *D. longicatena* (Dl), (C) *Lachnospiraceae* 24 430 (Lachno), (D) *O. acetigenes* (Oa), (E) *C. difficile* (Cd), (F) *B. adolescentis* (BA), (G) *B. longum* (BL), (H) *B. thetaiotaomicron*, and (I) *B. uniformis* with SGH10 and mutants deficient in the synthesis of colibactin, mccE492, or both under anoxic conditions; Dunnett’s multiple comparisons test was performed on CFU values to determine differences in means; ^*^ denotes *P* < .05 and ^**^ denotes *P* < .01.

Although mccE492 is not required for gut colonization, it is beneficial for HvKp to express both mccE492 and colibactin in the gut. *E. coli* was killed by SGH10 under oxic and anoxic conditions but it did not affect *K. pneumoniae* growth (Fig. 6A, Supplementary Fig. 5). Deletion of both GIE492 and ICEKp10 restored survival of *E. coli* (Fig. 6A). When competed with SGH10Δ*mceA* or SGH10Δ*clbP*, more *E. coli* survived relative to SGH10, showing that mccE492 and colibactin kill *E. coli* through independent pathways (Fig. 6A). Additionally, more *E. coli* survived when competed with SGH10Δ*mceA*Δ*clbP* than with SGH10Δ*mceA* or SGH10Δ*clbP* under oxic conditions (Fig. 6A). Although the GIs may contain other factors that enable SGH10 to kill *E. coli* or scavenge nutrients such as iron, these results suggest that mccE492 and colibactin cooperate to enhance the effect of colibactin.

Complementation of SGH10Δ*clbP* and SGH10Δ*mceA*Δ*clbP* with wildtype ClbP but not the catalytically inactive ClbP^S95A^ resulted in increased killing of *E. coli* (Supplementary Fig. 6A), confirming the effects due to active colibactin. To demonstrate the specificity of killing, we expressed their cognate immunity proteins MceB and ClbS in *E. coli*. *E. coli* carrying control vectors was susceptible to killing by mccE492 and colibactin (Supplementary Fig. 6B). When *E. coli* expressing *mceB* was competed with SGH10 and SGH10Δ*clbP*, no difference in survival was observed due to protection against microcin. However, colibactin-specific killing could be observed when *E. coli* expressing *mceB* were competed with SGH10Δ*mceA* or SGH10Δ*mceA*Δ*clbP* (Supplementary Fig. 6B). When *clbS* was expressed in *E. coli*, only microcin-dependent killing was observed. When both immunity proteins were expressed, there were no differences in survival when competed with SGH10 or all mutants, indicating that the immunity proteins could protect *E. coli* against both forms of killing (Supplementary Fig. 6B). In these experiments, *E. coli* did not affect the growth of *K. pneumoniae* (Supplementary Fig. 6C and D).

ClbP has also been shown to be important in the export of microcins in *E. coli* Nissle [42]. To rule out the possibility that our microcin and colibactin mutants possess defects in other pathways, we assessed the amounts of mccE492 of SGH10 and SGH10Δ*clbP* (Supplementary Fig. 6E). There were no differences in mccE492 activity between the strains, and so mccE492 export is independent of ClbP.

We then competed HvKp with obligate anaerobes known to colonize the human gut. Intriguingly, we found that the human gut obligate anaerobes *Dorea longicatena*, *Lachnospiraceae* 24430, and *Oscillibacter acetigenes* were susceptible to the effects of colibactin (Fig. 6B–D). These human gut isolates correspond to the taxa from the *Clostridiales* order that were depleted in mice colonized with colibactin-producing SGH10 in Fig. 5C. Other important human gut anaerobes such as *Clostridioides difficile*, *Bifidobacterium adolescentis*, and *Bifidobacterium longum* were also sensitive to mccE492 and colibactin (Fig. 6E–G).

**Figure 7:**
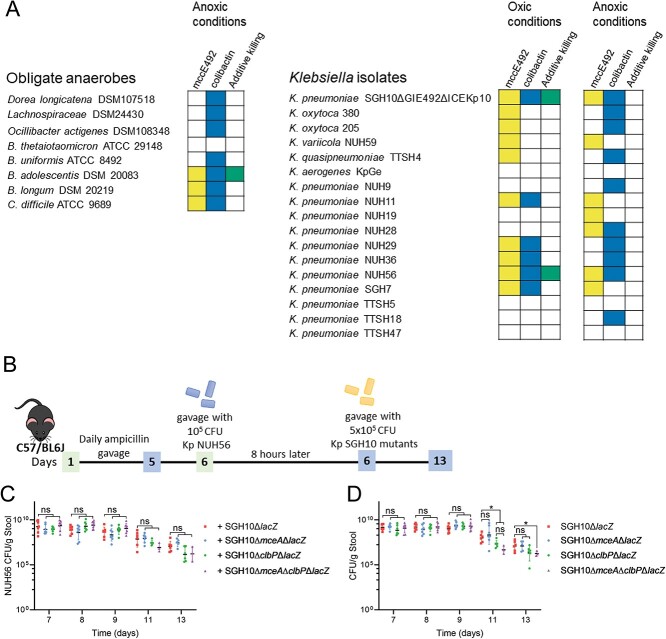
Effect of mccE492 and colibactin on other bacteria; (A) susceptibility of bacteria to mccE492 and colibactin; these bacteria did not possess immunity proteins to colibactin or mccE492; first column (yellow) denotes susceptibility to mccE492, second column (blue) denotes susceptibility to colibactin, and third column (green) denotes that a cooperative killing effect of both molecules is observed; unshaded boxes indicate insensitivity to these molecules; bacteria CFU are plotted in Fig. 6, and Supplementary Fig. 7,8; for all datasets described in this figure, mean ± SD are plotted (*n* = 3–4), and Dunnett’s multiple comparisons test was performed on CFU values to determine differences in means; ^*^ denotes *P* < .05 and ^**^ denotes *P* < .01; (B) an *in vivo* predator–prey experiment was conducted in the murine model of gastrointestinal colonization with *K. pneumoniae* (Kp) NUH56 as prey; C57BL6/J mice were infected with 10^5^ CFU of Kp NUH56 by oral gavage after ampicillin treatment for 5 days; 8 h later, the mice were gavaged with 5 x 10^5^ CFU of SGH10Δ*lacZ*, SGH10Δ*mceA*Δ*lacZ*, SGH10Δ*clbP*Δ*lacZ*, or SGH10Δ*mceA*Δ*clbP*Δ*lacZ*; (C) the bacterial loads of NUH56 or D the SGH10 Δ*lacZ* mutants in stool were quantified; the geometric mean and SD are plotted (*n* = 1), and Dunnett’s multiple comparisons test was performed on log-transformed CFU values to determine differences in means; ^*^ denotes *P* < .05.

**Figure 8:**
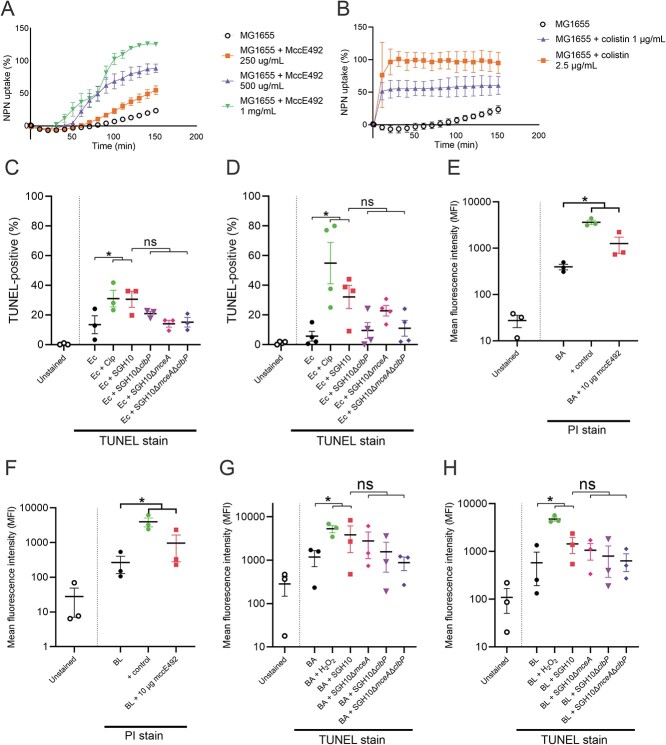
Mechanism of mccE492 and colibactin mediated killing of other bacteria; (A) outer membrane perturbation by mccE492 was measured by NPN uptake in *E. coli* MG1655; (B) colistin was used as a positive control for outer membrane perturbation; NPN assays were performed *n* = 3 times and values are expressed as mean ± SD; (C) the TUNEL assay was used to quantify DNA damage of *E. coli* MG1655 co-incubated with SGH10 and mutants on solid media for 4 h under oxic and (D anoxic conditions; 50 μg/ml ciprofloxacin (Cip) was used as a positive control for DNA damage; (E) Propidium iodide (PI) uptake was measured in BA and (F) BL treated with mccE492 for 30 min; a positive control (+ control) for PI uptake was generated by treating BA and BL with lysozyme, mutanolysin, and isopropanol; (G) TUNEL assays were conducted to measure DNA damage in BA and H BL co-incubated with SGH10 and mutants for 4 h under anoxic conditions; 1% H_2_O_2_ is used as a positive control for DNA damage, and mean fluorescence intensity (MFI) values were quantified; in this figure, mean ± SD are plotted (*n* = 3–4), and Dunnett’s multiple comparisons test was performed to determine differences in means; ^*^ denotes *P* < .05.

Additionally, we competed HvKp with a panel of *Klebsiella* isolates that were *clb*-*mce*- and *clbS*-*mceB*-. We have observed differences in the susceptibility of *E. coli* and *Klebsiella* species to mccE492 and colibactin during oxic or anoxic conditions (Figs 6A and 7A, Supplementary Figs 7 and 8). Some strains were sensitive to mccE492 under oxic conditions but these strains were not killed by mccE492 under anoxic conditions. Some strains insensitive to colibactin in oxic conditions were susceptible to colibactin during anaerobic growth. Only *K. pneumoniae* NUH28 and NUH56 were susceptible to both mccE492 and colibactin during anoxic conditions (Figs 7A and 8C). In these experiments, the growth of *K. pneumoniae* was not affected by co-culture with other bacteria (Supplementary Figs 9 and 10).

Our screen revealed that some Gram-positive bacteria such as *C. difficile*, *B. adolescentis*, and *B. longum* were susceptible to both colibactin and mccE492 (Figs 6E–G and 7A). The major Gram-negative human gut species *Bacteroides thetaiotaomicron* was resistant *K. pneumoniae* to both (Figs 6F and 7A). *Bacteroides uniformis* was susceptible only to colibactin (Figs 6I and 7A). Co-culture with anaerobes did not affect the growth of *K. pneumoniae* (Supplementary Fig. 11) In *E. coli* BW25113, *K. pneumoniae* NUH11 and NUH56, and *B. adolescentis*, the effect of microcin and colibactin together is greater than the effect of either factor alone (Fig. 6A, Supplementary Figs 7H and 8C).

### *K. pneumoniae* does not use mccE492 and colibactin to compete with closely related isolates

To determine whether mccE492 and colibactin in *K. pneumoniae* are important for competition with closely related bacterial prey, NUH56 was used as prey during *in vivo* competition (Fig. 7B) as it is a *mce*-*clb*-ST23 lineage strain that was susceptible to killing by colibactin and mccE492 (Fig. 7A). We did not observe statistically significant differences in the colonization of the NUH56 prey in competition with SGH10 or its various mutants (Fig. 7C).

We observed that expression of colibactin benefited SGH10 colonization even though it did not kill NUH56. Stool carriage of SGH10Δ*clbP*Δ*lacZ* and SGH10Δ*mceA*Δ*clbP*Δ*lacZ* was significantly reduced compared to SGH10Δ*lacZ* on Day 11 (Fig. 7D). On Day 13, stool carriage of SGH10Δ*mceA*Δ*clbP*Δ*lacZ* but not SGH10Δ*clbP*Δ*lacZ* was reduced relative to SGH10 (Fig. 7D). SGH10Δ*mceA*Δ*lacZ* colonized the gut as well as SGH10Δ*lacZ* (Fig. 7D). Stool bacterial loads of NUH56 were ~2 logs lower in mice colonized with SGH10Δ*clbP*Δ*lacZ* and SGH10Δ*mceA*Δ*clbP*Δ*lacZ* as compared to mice colonized with SGH10Δ*lacZ* and SGH10Δ*mceA*Δ*lacZ* on Day 13 (Fig. 7D). The expression of colibactin or colibactin and mccE492 is important for SGH10 to colonize even when a competing *K. pneumoniae* strain is present. These results support a model where the crucial targets of microcin and colibactin are not competitors closely related to HvKp such as other susceptible *K. pneumoniae,* but other members of the microbiome that affect colonization resistance. In fact, colibactin and mccE492 seem to even benefit the colonization of other *K. pneumoniae* strain such as NUH56 that do not produce these factors themselves.

### Effect of mccE492 and colibactin on bacterial prey

MccE492 is a pore-forming bacteriocin that exhibits activity against Gram-negatives [23, 24]. Using 1-N-phenylnaphthylamine (NPN) uptake as an output for outer membrane permeability, we demonstrated that purified mccE492 can perturb the outer membrane of *E. coli* in a dose-dependent manner (Fig. 8A and B). Moreover, purified mccE492 has a dose-dependent effect on the survival of the Gram-positives *B. adolescentis, B. longum*, and *C. difficile* (Supplementary Fig. 12).

Employing the TUNEL assay to examine DNA damage, we found that SGH10 induced a significant increase in TUNEL-positive *E. coli* under oxic and anoxic conditions (Fig. 8C and D, Supplementary Fig. 13). No significant increases in TUNEL-positive *E. coli* were observed when they were competed with SGH10Δ*mceA*, SGH10Δ*clbP*, or SGH10Δ*mceA*Δ*clbP* (Fig. 8C and D, Supplementary Fig. 13).

*C. difficile*, *B. adolescentis* (BA), and *B. longum* (BL) were sensitive to colibactin and mccE492 (Figs 6E–G and 7A). MccE492 has not been reported to have an effect against Gram-positives. However, it is possible that unmodified mccE492 can perturb their membranes. Using a propidium iodide (PI) uptake assay, we demonstrated that treatment with mccE492 increased permeability in BA and BL (Fig. 8F and G, Supplementary Fig. 14). Although PI is not a specific assay for membrane perturbation, the NPN assay does not work with Gram-positives because the layer of peptidoglycan that shields the cytoplasmic membrane of Gram-positives is not hydrophobic, and NPN fluoresces in a hydrophobic environment such as in the Gram-negative outer membranes. These results show that mccE492 causes perturbation of membranes in BA and BL.

Additionally, we observed that SGH10 induced a significant increase in DNA damage in BA and BL (Fig. 8G and H, Supplementary Fig. 15). *K. pneumoniae*-induced DNA damage is dependent on both microcin and colibactin in both BA and BL (Fig. 8G and H). MccE492 appears to perturb the membrane of susceptible Gram-negative and Gram-positive bacteria. However, the genotoxic effect of colibactin on prey was observed only when both mccE492 and colibactin were expressed.

Colibactin can potentiate the death of susceptible bacteria by prophage induction [31]. We utilized the Δ9 strain of *E. coli* BW25113 that has been cured of all prophages [43] and found that it was still sensitive to colibactin, demonstrating that prophage induction is not necessary for colibactin-mediated killing of *E. coli* (Supplementary Fig. 16). Thus, *K. pneumoniae*-induced DNA damage was sufficient to kill *E. coli* without prophage induction. *K. pneumoniae* was unaffected by the presence of *E. coli* in these assays (Supplementary Fig. 16).

## Discussion

In this study, we demonstrate that GIs GIE492 and ICEKp10 are co-associated with the CG23-I and CG10118 HvKp lineages. The products of GIE492 and ICEKp10 play cooperative roles during gastrointestinal colonization of HvKp. Epistatic interactions such as co-selection can occur between genetic loci that possess complementary functions. ICEKp10 integration is linked to clonal expansion of the CG23-I subclade within ST23 [4], further supporting a model where a beneficial relationship exists between these two GIs. Indeed, we also observe convergent evolution in CG10118, another hypervirulent lineage distant from CG23 that has acquired ICEKp10, GIE492, and the *Klebsiella* virulence plasmid.

There is a strong, colibactin-driven benefit of expressing mccE492 and colibactin during colonization. Colibactin is not essential during systemic infection, which contrasts with previous work reporting that a *clbA* deletion mutant in the HvKp strain KP 1084 was attenuated in virulence [44]. This phenotype is likely due to pleiotropic effects of deleting ClbA on siderophore maturation [45]. Gastrointestinal colonization precedes translocation and development of disseminated infection. SGH10∆*clbP* was attenuated in lethality in our murine model of HvKp translocation, likely because it colonizes poorly, although colibactin-induced tissue damage could also enhance bacterial adhesion and translocation.

Many *K. pneumoniae* lineages have acquired GIE492, implying a benefit of producing mccE492. However, mccE492 is dispensable for HvKp SGH10 to colonize the murine gut. MccE492 could be more important in the context of competition in the environment rather than in the mammalian gut. Indeed, mccE492 has a strong effect on *E. coli* during oxic but not anoxic conditions. MccE492 alone did not cause significant shifts in the composition of murine gut microbiota, and mccE492 seems to play a supportive role to colibactin during colonization of *K. pneumoniae*.

Colibactin likely benefits gastrointestinal colonization because it alters the gut microbiome. In a murine model involving maternal to fetal transmission of colibactin-producing *E. coli*, colibactin was shown to be important for intraspecies competition with closely related *Enterobacterales* and *Firmicutes* [46]. In our model, colibactin-producing *K. pneumoniae* caused changes in the gut microbiome of adult mice, inhibiting several gut commensal taxa belonging to the *Clostridiales* order (*Lachnospiraceae* and *Christensenellaceae*) and *Muribaculaceae* (*Bacteroidales*). *Lachnospiraceae* are important in short chain fatty acid (SCFA) and bile acid production, and *Christensenellaceae* are associated with host metabolic health [47-49]. Inhibition of these taxa by colibactin could reshape the metabolic landscape of the gut to benefit *K. pneumoniae* colonization. We observed colibactin-dependent killing of *D. longicatena*, *Lachnospiraceae* 24430, and *O. acetigenes*, human gut commensal species that corresponded to the taxa inhibited in mice. These results corroborated our murine experiments. The human commensal isolates used have also been reported to produce SCFAs and modify host metabolism, further supporting the idea that inhibition of bacterial taxa by colibactin could affect metabolism in the gut [50-52].

Besides the above species, inhibition of human commensal taxa belonging to *Clostridiales* (*O. acetigenes*, *Lachnospiraceae* 24430) and *Bacteroidales* (*B. uniformis*) order further bolsters the possibility that HvKp could be in competition with commensal species in the gut. SGH10 did not use colibactin and mccE492 to kill a closely related susceptible prey strain NUH56 in the gut. These factors may only provide a slight competitive advantage against closely related *Enterobacterales* bacteria perhaps because of spatial segregation. Our results strongly suggest that colibactin has a greater effect on gut commensals. The benefit for HvKp SGH10 inhibiting NUH56 is likely outweighed by the advantage of SGH10 killing gut commensals that compete with NUH56, overall benefiting both *K. pneumoniae* strains. The main target of colibactin appears to be gut commensal species in closer proximity to the colonizing strain, and the specificity of colibactin is not as narrow as initially imagined.

We made the unexpected discovery that Gram-positive anaerobes were susceptible to mccE492 because mccE492 is thought to be restricted in activity to related Gram-negative bacteria expressing catechol siderophore receptors. Perhaps, mccE492 can insert into the membrane of Gram-positives through a siderophore-independent mechanism. MccE492 can insert into artificial liposomes and lipid bilayers and form pores [21]. We observed a cooperative killing effect of colibactin and mccE492 in a small number of bacteria. Moreover, *K. pneumoniae*-induced DNA damage in *E. coli*, *B. adolescentis*, and *B. longum* is dependent on the presence of mccE492 and colibactin. The mechanism by which colibactin enters mammalian or bacterial cells is still unknown. We hypothesize that during cooperative killing of prey, mccE492 disrupts the cellular envelope and enhances uptake of colibactin. Alternatively, mccE492 could cause DNA damage in bacterial prey as it has been shown to induce apoptosis in Hela cells [53]. Moreover, these observations are supported by *in vivo* gastrointestinal colonization where mccE492 alone is dispensable for colonization but enhances the beneficial effect of colibactin.

In summary, we describe a co-association between GIE492 and ICEKp10 in two HvKp lineages. The combination of colibactin and mccE492 is beneficial during colonization of HvKp in the gastrointestinal tract. Colibactin and mccE492 enable HvKp to kill bacterial gut commensals to gain a foothold in the crowded endogenous microbiome space. Our results suggest that the benefits that GIE492 and ICEKp10 confer during colonization have contributed to the dominance of the CG23-I lineage.

## Supplementary Material

Supplementary_Information_15_Mar_2024_wrae054

Supplementary_Table_1_wrae054

Supplementary_Table_2_wrae054

Supplementary_Table_3_wrae054

Supplementary_Table_4_wrae054

Supplementary_Table_5_wrae054

Supplementary_Table_6_wrae054

Supplementary_Table_7_wrae054

## Data Availability

The complete genome sequence of *K. pneumoniae* RYC492 was deposited under the Bioproject accession PRJNA179092. *K. pneumoniae* genome assemblies from the A-KLASS study have been deposited in GenBank under BioProject PRJNA956314. The complete sequences of GIE492 variants and *mce* alleles have been made available in Supplementary Data 1. Supplemental methods are available in Supplementary Information.
