# Correction to: Hypervirulent *Klebsiella pneumoniae* employs genomic island encoded toxins against bacterial competitors in the gut

**DOI:** 10.1093/ismejo/wrae143

**Published:** 2024-08-13

This is a correction to:

Yi Han Tan, Patricio Arros, Camilo Berríos-Pastén, Indrik Wijaya, Wilson H W Chu, Yahua Chen, Guoxiang Cheam, Ahmad Nazri Mohamed Naim, Andrés E Marcoleta, Aarthi Ravikrishnan, Niranjan Nagarajan, Rosalba Lagos, Yunn-Hwen Gan, Hypervirulent *Klebsiella pneumoniae* employs genomic island encoded toxins against bacterial competitors in the gut, *The ISME Journal*, Volume 18, Issue 1, January 2024, wrae054, https://doi.org/10.1093/ismejo/wrae054

In the originally published version of this manuscript, Figure 7B depicted on day 6 the wrong prey species as the Kp SGH10 GIE492ICEKp10 double deletion mutant. It should be Kp NUH56 instead. Furthermore, the interval should not be 24h but 8h before the gavage with Kp SGH10 mutants.

These errors have been corrected in the article.

