# Supplementary material for: Hypervirulent Klebsiella pneumoniae employs genomic island encoded toxins against bacterial competitors in the gut
Source: ISME J. 2024 Mar 28;18(1):wrae054. doi: 10.1093/ismejo/wrae054 (PMC11020217; doi:10.1093/ismejo/wrae054)
Supplement: Supplementary_Information_15_Mar_2024_wrae054 [file supplementary_information_15_mar_2024_wrae054.docx]

**List of Supplementary Tables**

**Supplementary Table 1. List of bacteria strains used in this study**

**Supplementary Table 2. Main features of the *K. pneumoniae* genomes analysed in this study.**

**Supplementary Table 3. Differences in α-diversity between mice colonised with SGH10 or mutants**

**Supplementary Table 4. Differential abundance analysis of microbial taxa**

**Supplementary Table 5. List of primers used in this study**

**Supplementary Table 6. Mapping of reads**

**Supplementary Table 7. Selection strategies used to differentiate between *K. pneumoniae* and competing bacterial strains**

**Supplementary Data 1. Allele sequences of GIE492 variants**

**Supplemental Methods**

***K. pneumoniae* genome database construction and phylogenomic analyses**

17,612 *K. pneumoniae* species complex (KpSC) genomes were downloaded from the NCBI RefSeq database on April 5th, 2023. Upon filtering out genomes of poor quality with ambiguous nucleotide bases and non-standard *Klebsiella* genomes, 11,817 genomes were selected for further analysis. We included *K. pneumoniae* RYC492, 119 genomes from the Antibiotics for Klebsiella Liver Abscess (A-KLASS) cohort ^1^, 365 *K. pneumoniae* isolates from a bloodstream infection cohort (BSI) ^2^, 34 *Klebsiella* genomes from the Murray Collection ^3^ and 97 CG23 genomes from Lam et al 2018^4^. The resulting 12,433 *K. pneumoniae* *sensu stricto* species genomes set was screened with a multilocus sequence typing (MLST) approach for ICE*Kp* variants and other features using Kleborate v2.3.2 ^5^.

For the GIE492+ *K. pneumoniae* phylogenomic analyses, we used a core genome MLST (cgMLST) approach based on the 629 loci scgMLSTv2 scheme and dual barcoding method of Hennart et al ^6^. Allele search was performed using BLASTn v2.13.0, with 95% identity and 95% coverage thresholds. First, the best hit for each allele was chosen according to the bitscore. Then, the selected alleles were used to generate cgMLST profiles for each genome, which along with 34,055 reference profiles, were used as input for the LINcoding algorithm to assign a cgLIN code to each genome ^6^. Then, the assigned cgLIN codes were used to identify clonal groups and sublineages. Finally, a maximum-likelihood tree was inferred from a core genome multilocus sequence alignment (cgMSA) including 588 non-redundant GIE492+ genomes (differing in at least one allele). The cgMSA was generated using MAFFT ^7^ in the globalpair mode. The tree was inferred with IQ-tree ^8^ with the GTR+F+I+R4 substitution model, selected upon Model Finder Plus (MFP) analysis ^9^, and 10,000 UFBoostraps ^10^.

**GIE492 typing**

The *K. pneumoniae* genome set was screened for all 23 genes of GIE492 described originally in *K. pneumoniae* RYC492 and its putative transfer origin (oriT) ^11^ using BLASTn ^12^ with 95% identity and coverage thresholds. A similar strategy was used to search for GIE492 among the 82,206 *Enterobacterales* genomes (taxID:91347) available from RefSeq on May 26^th^, 2023. Additionally, the genomes were screened for *clbS*, using reference sequences from *E. coli* (Genbank KX683217.1), *Citrobacter koseri* (NC_009792.1), and *Frischella perrara* (NZ_CP009056.1). The RYC492 genome was sequenced via Illumina and Nanopore and the complete genome was assembled using a hybrid assembly strategy based on the Trycycler tool ^13^. Genome completeness and contamination were assessed using checkM ^14^. The best hit was chosen based on the bitscore value.

For GIE492 typing, the alleles for the 23 genes were identified and compared among the strains, and allele sequences for GIE492 typing are provided as Supplementary Data 1. The representative sequence of each GIE492 variant was selected among the less fragmented genomes according to N50, contig number, and largest contig length: Type I: GCF_904864645.1; II: GCF_012952475.1; III: GCF_904863285.1; IV: GCF_900506655.1; V: GCF_020526065.1; VI: GCF_002866675.1; VII: GCF_900775005.1; VIII: GCF_003660185.1. The two *K. michiganensis* GIE492 variants were found in the assemblies NZ_JNCH01000034 and NZ_CP106658. The schema for GIE492 variant alignment was made using EasyFig ^15^. The GIE492 variants' sequence alignment was made using MAFFT in the globalpair mode ^7^. Then, the derived distance tree was inferred using IQ-tree with the K3Pu+F+Imodel and 10,000 UFBootstraps ^8^.

**Construction of deletion mutants in bacteria**

All primers used in this paper are listed in Supplementary Table 5. Markerless deletion mutants were generated in SGH10 for ICEKp10 (bases 1782875 - 1920668), GIE492 (bases 1922546 - 1944844), *mceA* (SGH10_RS09405), *clbP* (SGH10_RS09005), and *iucCD* (SGH10_RS27940, SGH10_RS27945) in SGH10. A conditional suicide vector was generated as described ^16^, and conjugated into SGH10 using *E. coli* S17-1λpir as a donor. Subsequently, negative selection was performed by growing SGH10 with the pR6KmobsacB vector in LB with 20% sucrose (Sigma-Aldrich). Deletion mutants were screened using PCR and whole genome sequencing to validate gene deletion. Double and triple deletion mutants were generated by introducing the respective deletion construct into a single mutant or double mutant. SGH10Δ*lacZ* has been described ^16^, and other Δ*lacZ* mutants in this study were constructed using the deletion vector described in this study.

**Complementation of bacterial mutants**

Full length *clbP* was amplified from genomic DNA of SGH10 and ligated into the cut pMLBAD plasmid to make pMLBAD-*clbP* using the NEBuilder HiFi DNA Assembly Master Mix. To generate *clbP*^S95A^, *clbP* was amplified in two fragments and the asparagine residue was substituted for an alanine using a forward primer. The fragments were assembled into the cut pMLBAD plasmid to give the pMLBAD- *clbP*S95A plasmid using NEBuilder HiFi DNA Assembly Master Mix. *clbS* was amplified from genomic DNA of SGH10 and cloned into the pUCP28T (abbreviated as p28T) vector, and constitutively expressed under the control of the Plac promoter.

pACYC184-catR and pACYC184-catR-*mceB* were derived from the pBAML vector. The pBAML vector consists of a pACYC184-catR (abbreviated as p184) backbone expressing *mceAB* under the control of the native promoter. We generated pACYC-catR by amplifying the plasmid backbone from pBAML without *mceAB*. The resultant linear fragment was annealed using the NEBuilder® HiFi DNA Assembly Master Mix to give pACYC-catR. As *mceB* is translated before *mceA*, we amplified a linear fragment of pACYC184-catR without *mceA* from pBAML. This fragment was then annealed into a circular plasmid using the NEBuilder HiFi DNA Assembly Master Mix to give pACYC184-catR-*mceB*.

**Microcin agar diffusion assays**

Overnight cultures of *K. pneumoniae* and *E. coli* MG1655 prey strains were grown in LB + 100 µM 2,2"-Bipyridyl (DIP) (Sigma Aldrich). 5×10^7^ CFU/mL *E. coli* prey was prepared in molten LB + DIP + 0.3% Bactoagar (1^st^ BASE Asia), and 5 mL of this mixture was poured onto LBA + DIP plates to give a soft agar overlay. 1 µL of 10^9^ CFU/mL *K. pneumoniae* predator cultures was spotted onto the agar overlay, and the plates were incubated at room temperature for 16 h before being imaged.

To measure secreted mccE492 antibacterial activity, overnight cultures of SGH10 and mutants were grown in DMEM + 10%FBS, and diluted to OD 2. Bacterial cells were pelleted, and the supernatant was filtered through 0.22 µM filters. Then, 4 µL drops of 2-fold serial dilutions in water of the filtered supernatants were spotted on a soft agar lawn (LB + 0.3% Bacto agar) containing 5×10^7^ CFU/mL of the *E. coli* MG1655 prey. Plates were incubated at room temperature for 16h before being imaged to detect growth inhibition halos.

**Mammalian cell culture**

Mammalian cells were maintained in a CO_2_ incubator (ThemoFisher Scientific) at 37°C with 5% CO_2_. HepG2 cells (ATCC) were maintained in DMEM supplemented with 10% FBS (Singlab) and 1× penicillin–streptomycin (Singlab).

**Infection of mammalian cells**

50,000 HepG2 cells were seeded in the wells of a 24-well plate (Corning). Cells were infected with SGH10 and mutants that had been opsonised with 50% human serum for 30 min at an MOI of 50:1. At 4 h post infection (hpi), old media was removed from the wells and the cells were washed with 1×PBS before 1 mL of DMEM + 10% FBS + 500 µg/mL kanamycin was added to kill extracellular bacteria. At 48 hpi, the cells were washed with 1 mL 1×PBS to remove extracellular bacteria and fixed with 4% formaldehyde (Sigma-Aldrich) for 20 min. The wells were then stained with Giemsa stain or methylene blue (Sigma-Aldrich) for visualisation of megalocytotic cells.

For western blot, HepG2 cells were seeded and infected as above and cells were harvested at 8 hpi. 200 µg of cytarabine (Sigma-Aldrich) was used as a positive control. For acid extraction of histones, cells were lysed in 0.5%Triton X-100 + 1×PBS + 1X Protease Inhibitor (Pierce) on ice for 10 min. Nuclei were pelleted at 6,500g for 10 min at 4°C. The supernatant was used for the cytosolic fraction, and the pellet was resuspended in 0.2 N HCl at a density of 4×10^7^ nuclei per mL for acid extraction overnight at 4°C. Samples were then subjected to western blotting using antibodies for GAPDH (sc-32233, Santa Cruz Biotechnology), γH2AX (2F3, Biolegend) and H2AX (PA1-41004, Invitrogen), and the secondary antibodies HRP-linked anti-mouse antibody (559626, BD Pharmingen) and HRP-linked anti-rabbit antibody (7074, Cell Signalling Technologies).

**Microbiome analysis methods**

Raw reads were filtered to remove low quality bases and adapter sequences were removed using fastp (v0.20.0, default parameters) ^17^. Mouse host reads were removed by mapping to the GRCm39 reference using BWA-MEM (v0.7.17-r1188, default parameters) ^18^ and samtools (v1.9) ^19^. The remaining reads were classified using Kraken2 (v2.0.8) ^20^ with the Mouse Gastrointestinal Bacteria Catalogue (MGBC) database ^21^ containing mouse bacterial genomes. Read counts can be found in Supplementary Table 6. Operational taxonomic unit (OTU) abundance was estimated using Bracken (v2.5, r=100) ^22^. Species with maximum relative abundance <0.1% were removed to reduce false-positive calls and the relative abundances for the remaining species were renormalised to sum to 1. Reads are deposited in the EMBL Nucleotide Sequence Database (ENA) under project id PRJEB64247.

Alpha diversity (Simpson Index) and beta diversity (Bray-Curtis distance) were calculated using the vegan R package (v2.6-2) ^23^. Linear mixed-effect models with the different groups as fixed effects and the batch information as random effects were used. The models were built for each comparison with the wildtype group separately, and the alpha value cutoff was set to 0.05. PERMANOVA analysis was performed on the Bray-Curtis data using ‘adonis2’ from the vegan R package (v2.6-2). Plots were drawn using the ggplot2 R package ^24^. For differential abundance analysis, *K. pneumoniae* reads and low abundance taxa were removed by considering only taxa with relative abundance >0.1% and corresponding prevalence >25% for each set of pairwise comparisons. Analysis between different groups were done using Microbiome Multivariable Association with Linear Models (MaAsLin2) (v1.8.0) ^25^ which accounts for batch variables as random effects. *P* values were adjusted for multiple test correction using the Benjamini-Hochberg method.

***In vitro* competition assays**

Specific conditions for competition of bacteria are listed below, and strategies for differentiating *K. pneumoniae* predator and prey bacteria can be found in Supplementary Table 7. Overnight cultures of aerobic bacteria were grown under oxic conditions in DMEM + 10% FBS and anaerobic bacteria were grown in RCM in anoxic conditions.

For competition of *K. pneumoniae* SGH10 mutants and *E. coli*, *E. coli* cultures were diluted to 10^8^ CFU/mL, and *K. pneumoniae* cultures were diluted to 10^7^ CFU/mL in DMEM + 10% FBS, and equal volumes of these suspensions were mixed to give a ratio of 10:1 *E. coli* to Kp. The *E. coli* BW25113 and Δ9 strain were transformed with pACYC184 carrying the chloramphenicol resistance marker.

For competition of *K. pneumoniae* SGH10 mutants with other *Klebsiella* isolates, bacterial cultures were diluted to 10^8^ CFU/mL. Equal volumes of these suspensions were mixed to give a ratio of 1:1 and the bacteria were competed for 24 h.

For competition of *K. pneumoniae* SGH10 mutants with *C. difficile*, *C. difficile* cultures were diluted to 10^8^ CFU/mL in pre-reduced RCM, and *K. pneumoniae* cultures were diluted to 10^7^ CFU/mL in DMEM + 10% FBS, and equal volumes of these suspensions were mixed to give a ratio of 10:1 *C. difficile* to Kp. The bacteria were competed for 24 h.

For competition of *K. pneumoniae* SGH10 mutants with *Bacteroides thetaiotaomicron, Bacteroides uniformis, Bifidobacterium longum*, *Bifidobacterium adolescentis*, *Dorea longicatena*, *Lachnospiraceae* 24430, and *Oscillibacter acetigenes* anaerobic cultures were diluted to 5x10^8^ CFU/mL. *K. pneumoniae* cultures were diluted to 5x10^6^ CFU/mL, and equal volumes of these suspensions were mixed to give a ratio of 100:1 prey to *K. pneumoniae*. *Bacteroides* were competed with *K. pneumoniae* for 72 h and the other taxa were competed with *K. pneumoniae* for 24 h.

20 µL of the mixed bacterial suspensions was spotted on a cellulose nitrate 0.45 µM filter (Sartorius) placed on a DMEM agar plate. The plates were then placed in a 37°C bacterial incubator under oxic or anoxic conditions. Subsequently, filters were transferred from the agar plate into 2 mL of 1XPBS to resuspend bacteria and appropriate dilutions of bacterial suspension in 1XPBS or reduced RCM were plated. Where appropriate, bacteria were plated on LBA spread with 1 mM IPTG and 20 mg/ml X-gal for blue-white colony selection and appropriate dilutions of bacterial suspension in 1×PBS were plated.

**Purification of mccE492**

MccE492 was purified as described previously ^26^. In brief, mccE492 was expressed in overnight cultures of *E. coli* BL21(DE3) ^27^. Culture supernatant was filtered through 0.22 µm pore Stericup filters (Merck) and mixed with Bondapak C18 resin (Waters) to bind the microcin and incubated at 4°C for 2 h. The resin-bound mccE492 was then washed with 40% methanol and eluted in a step-wise acetonitrile (ACN)/water gradient. Subsequently, the peptide was dialysed against nanopure water and then lyophilized. Peptides were stored at -20°C and reconstituted in distilled water. Each mccE492 preparation was checked for activity using an agar diffusion assay before experimental use.

**1-N-phenylnaphthylamine (NPN) uptake assay**

Log cultures of *E. coli* MG1655 were grown in LB. Bacterial cells were pelleted and resuspended in 5 mM HEPES buffer (pH 7.2) containing 20 mM glucose to an OD 600 of 1. 100 μL of cells were added to 50 μl of 40 μM 1-N-phenylnaphthylamine (NPN) (Sigma Aldrich) in the above buffer in each well of black clear-bottom 96-well plates (Corning). Subsequently, 50 μl of the same buffer containing either MccE492 or colistin sulfate (Sigma Aldrich) was added to each well. Fluorescence was measured at an excitation wavelength of 355 ± 5 nm and emission wavelength of 420 ± 5 nm in a Tecan® infinite M1000 Pro every 10 min for 150 min.

**MccE492 killing assays**

Overnight cultures of *C. difficile*, *B. adolescentis* and *B. longum* were grown in RCM. For *B. adolescentis* and *B. longum*, bacterial cultures were diluted to 5×10^6^ CFU/mL and *C. difficile* cultures were diluted to 10^7^ CFU/mL in pre-reduced 1×PBS. 10 µL of the resultant bacterial culture was mixed with 10 µL of mccE492 which had been prepared in 1×PBS. This preparation was spotted onto one sixth of a cellulose nitrate 0.45 µM filter (Sartorius) on an RCM agar plate, and bacteria were subsequently incubated at 37°C for 6 h under anoxic conditions. Bacterial CFU were enumerated by resuspending bacteria on the filter into 1×PBS and plating appropriate dilutions on RCM agar.

**Terminal deoxynucleotidyl transferase dUTP nick end labeling (TUNEL) Assay**

*E. coli* was competed with SGH10 and other mutants at a ratio of 10:1 *E. coli* to *K. pneumoniae* on a DMEM agar plate under oxic or anoxic conditions. *B. adolescentis* and *B. longum* were competed with SGH10 and other mutants in a ratio of 10:1 on an RCM agar plate for 4 h under anoxic conditions.

A pACYC-mApple-chlR plasmid was used to tag *K. pneumoniae* with a mApple fluorescent reporter. After competition, bacteria were fixed in 1% formaldehyde for 20min on ice. Gram-negative and Gram-positive bacteria were stored in 70% ethanol and 70% isopropanol respectively at -20°C. The following day, bacteria were permeabilized with 0.1% Triton-X20 (Sigma-Aldrich) + 0.1% sodium citrate (Sigma-Aldrich) in 1×PBS for 2 min on ice and stained with the TUNEL click it kit (Invitrogen). The stained bacteria were subjected to flow cytometry on a Becton-Dickinson Fortessa. TUNEL staining was captured on the Alexa488 channel and mApple signal was captured on the mCherry channel. *K. pneumoniae* was excluded by gating only the mCherry-negative cell population and analysing the Alexa-488 staining in *E. coli*, *B. adolescentis* and *B. longum*.

**Propidium Iodide uptake assays**

Overnight cultures of *B. adolescentis* and *B. longum* were pelleted and resuspended in pre-reduced 1×PBS at a concentration of 1×10^9^ CFU/mL. 10 µL of the resultant bacterial culture was mixed with 10 µL of mccE492 prepared in 1×PBS. This preparation was spotted onto a cellulose nitrate 0.45 µM filter (Sartorius) on an RCM agar plate. The plate was subsequently incubated at 37°C for 30 min under anoxic conditions before the filters were transferred into 300 µL of 0.85% NaCl + 7.5 µM propidium iodide. The tubes were vortexed vigorously to resuspend bacteria into the PI staining buffer. Bacteria were incubated for 20 min on ice before flow cytometric quantification. Positive controls were generated by treating bacterial cultures with 3 U/mL mutanolysin (Sigma-Aldrich) and 2mg/mL lysozyme (Sigma-Aldrich) for 6 h, followed by 70% isopropanol for 1 h.


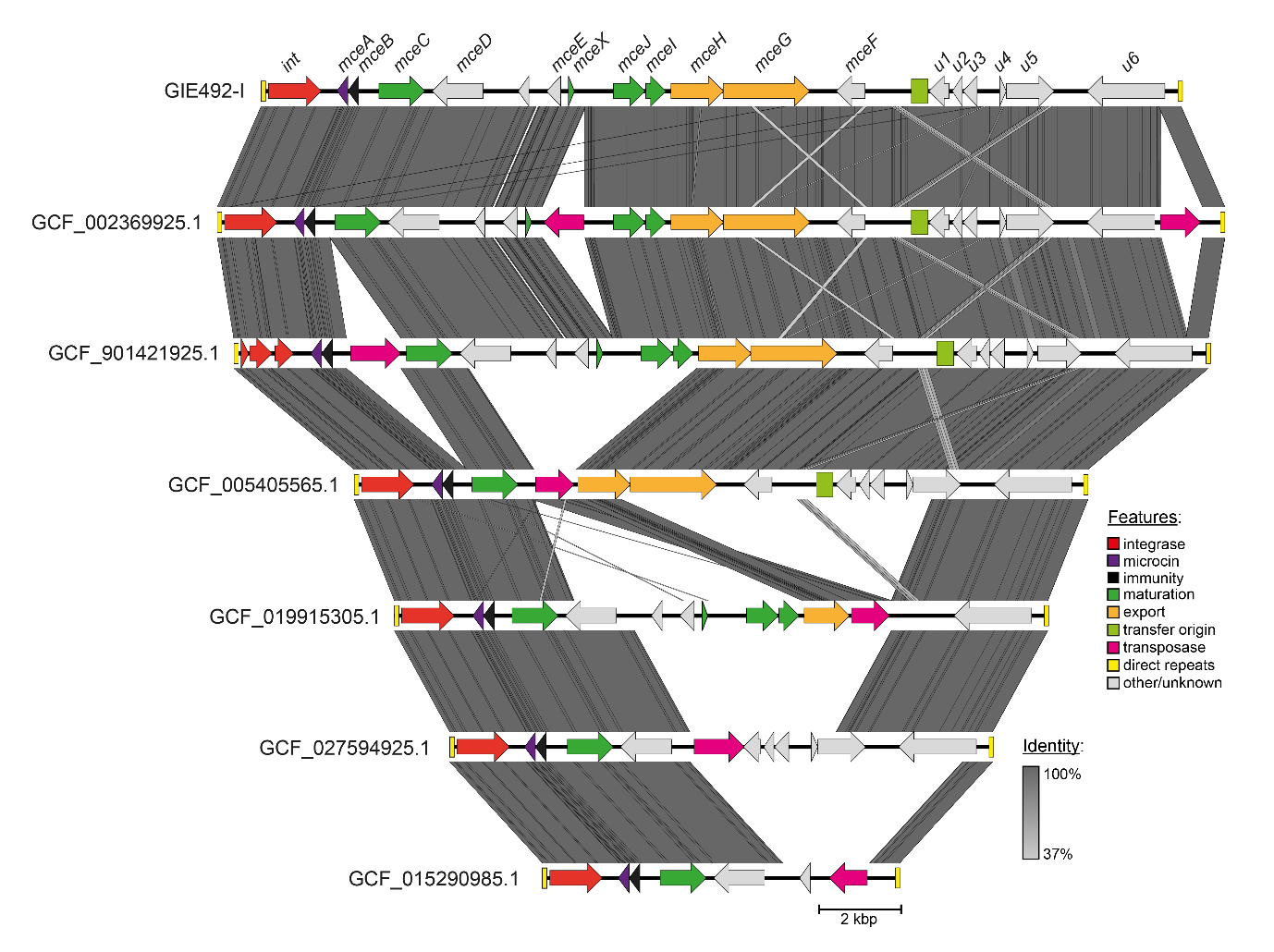


Supplementary Figure 1. GIE492 variants bearing deletions or insertion sequences found with low prevalence in *K. pneumoniae*. The GIE492-I variant was included as reference, and the accession numbers of the assembly for each variant is indicated. GCF_901421925 has a frameshifted integrase-coding gene and the transposase genes depicted in pink correspond to ISKpn72 or ISKpn74.


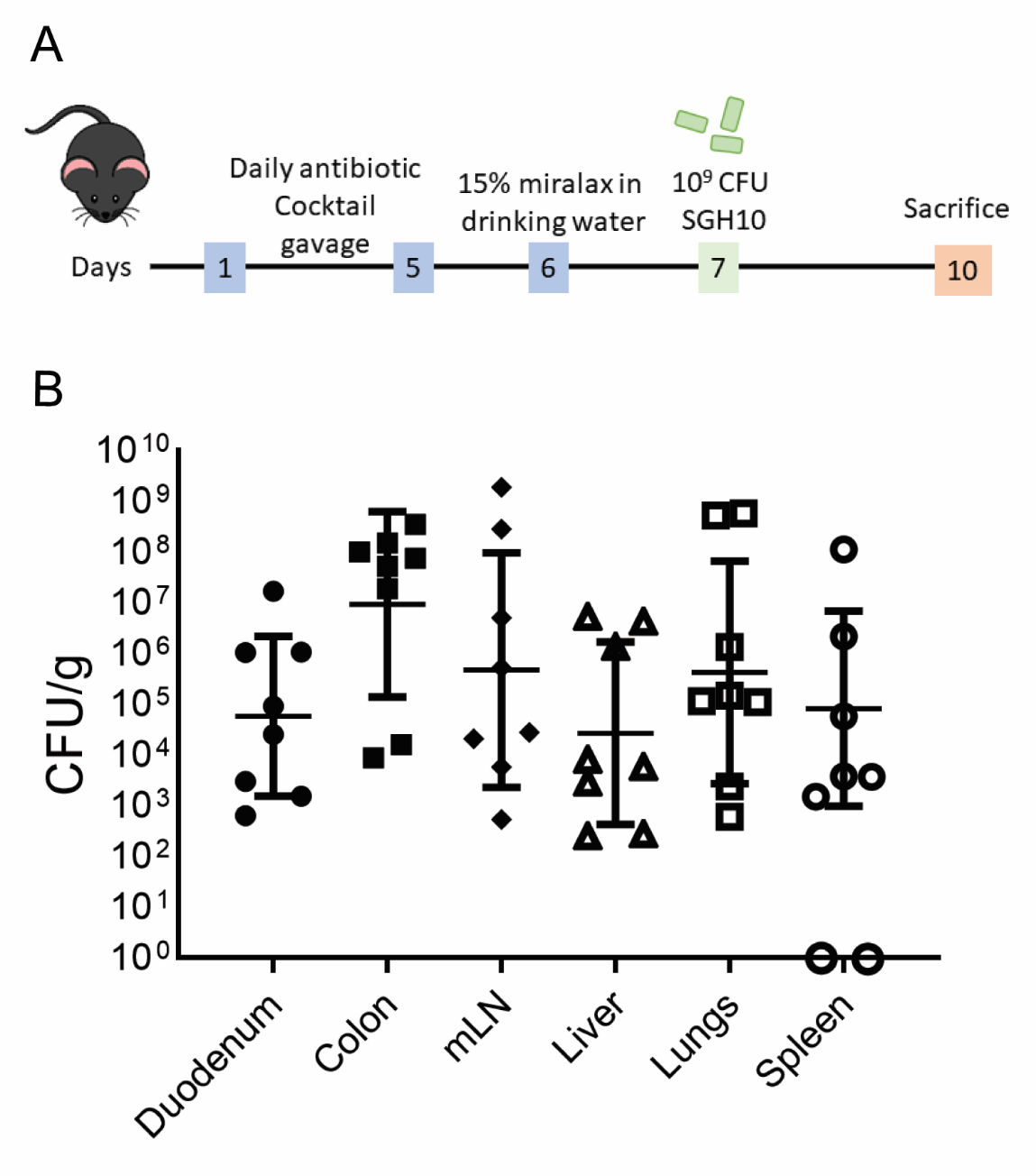


Supplementary Figure 2. A murine model of HvKp translocation. A Translocation of SGH10 was established by treating C57BL6/J mice with an antibiotic cocktail for 5 days prior to supplementation of drinking water with 15% Miralax. Subsequently, the mice were infected with 10^9^CFU SGH10 by oral gavage. B Mice were sacrificed when they met termination criteria and bacterial load was quantified in the organs of these mice. Geometric means and SD are plotted (n=1).


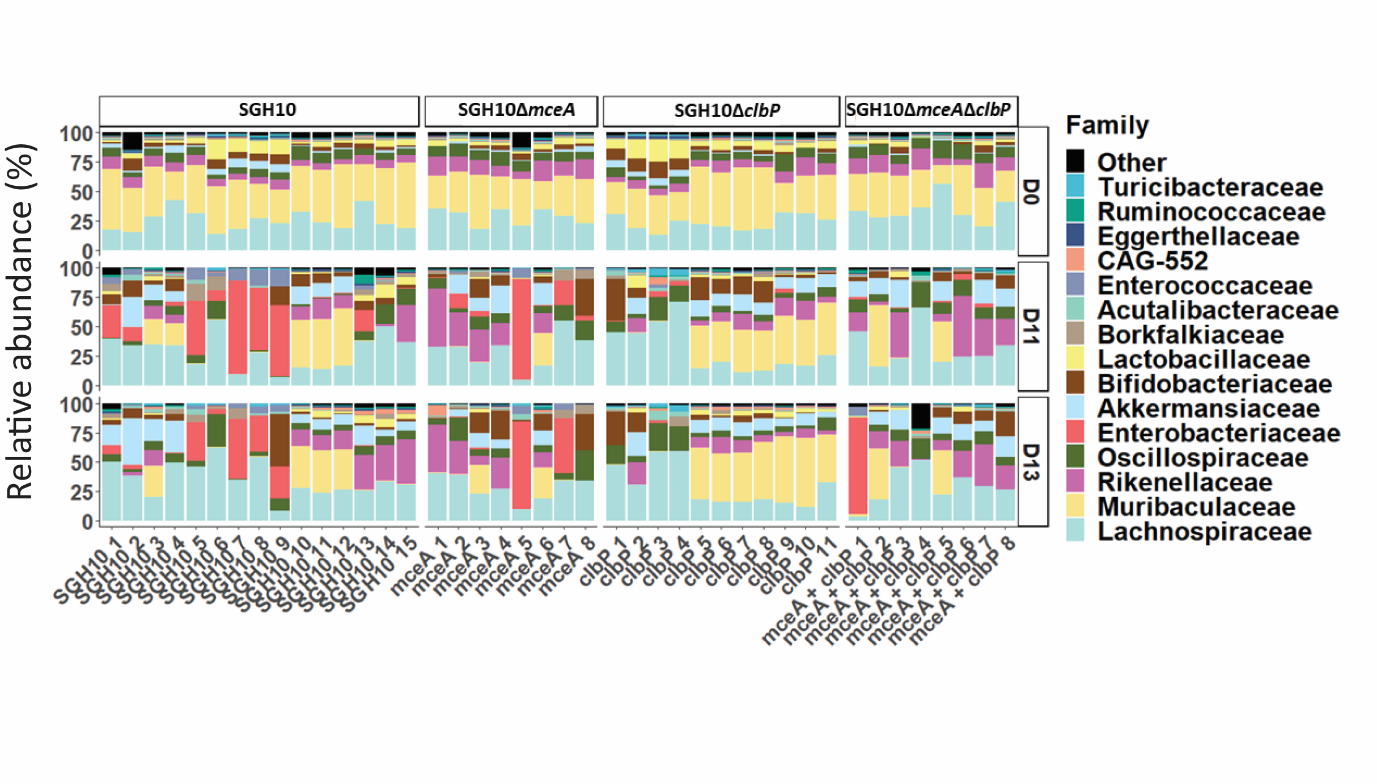


Supplementary Figure 3. Relative abundance of microbial taxa at the rank of Family


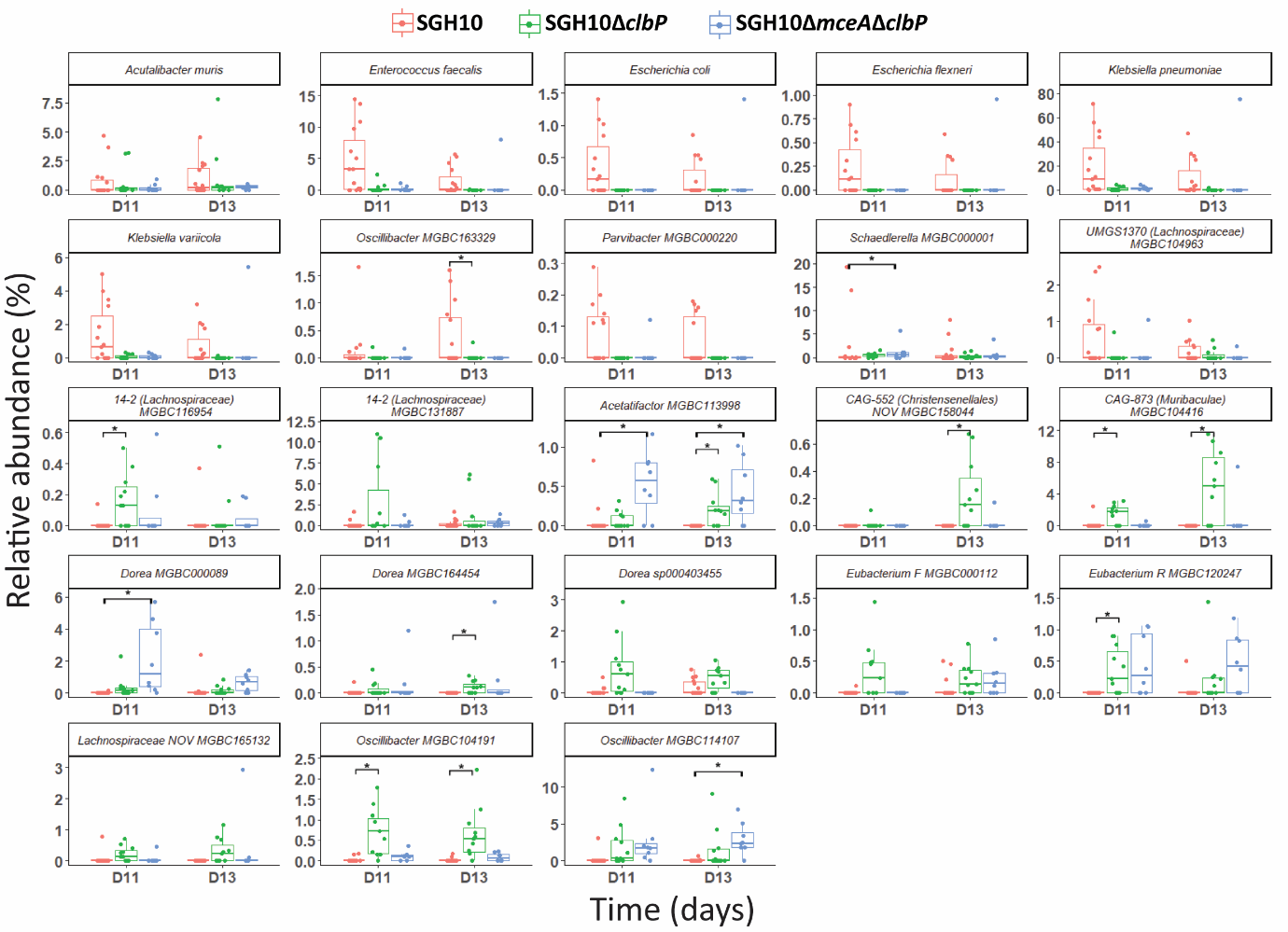


Supplementary Figure 4. Bracken plots of the relative abundance of taxa that are differentially enriched in mice colonised with wildtype SGH10 or mutant strains. *indicates *p* value < 0.05.


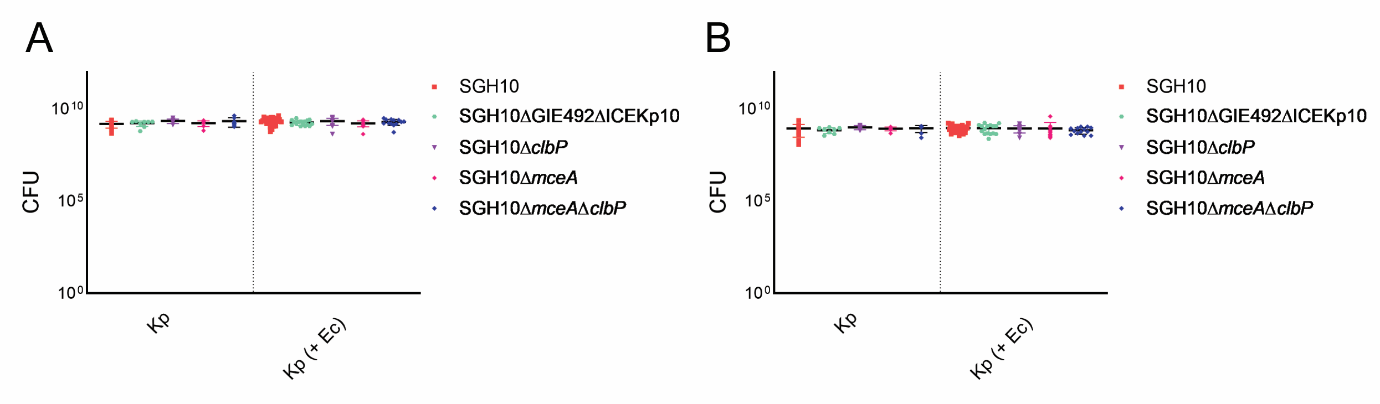


Supplementary Figure 5. The growth of *K. pneumoniae* co-cultured with *E. coli* BW25113Δ*lacY* under A oxic and B anoxic conditions for 24h. Mean ± SD of bacterial CFU is plotted (n=3). No significant differences in *K. pneumoniae* CFU were observed by Dunnett’s multiple comparisons test.


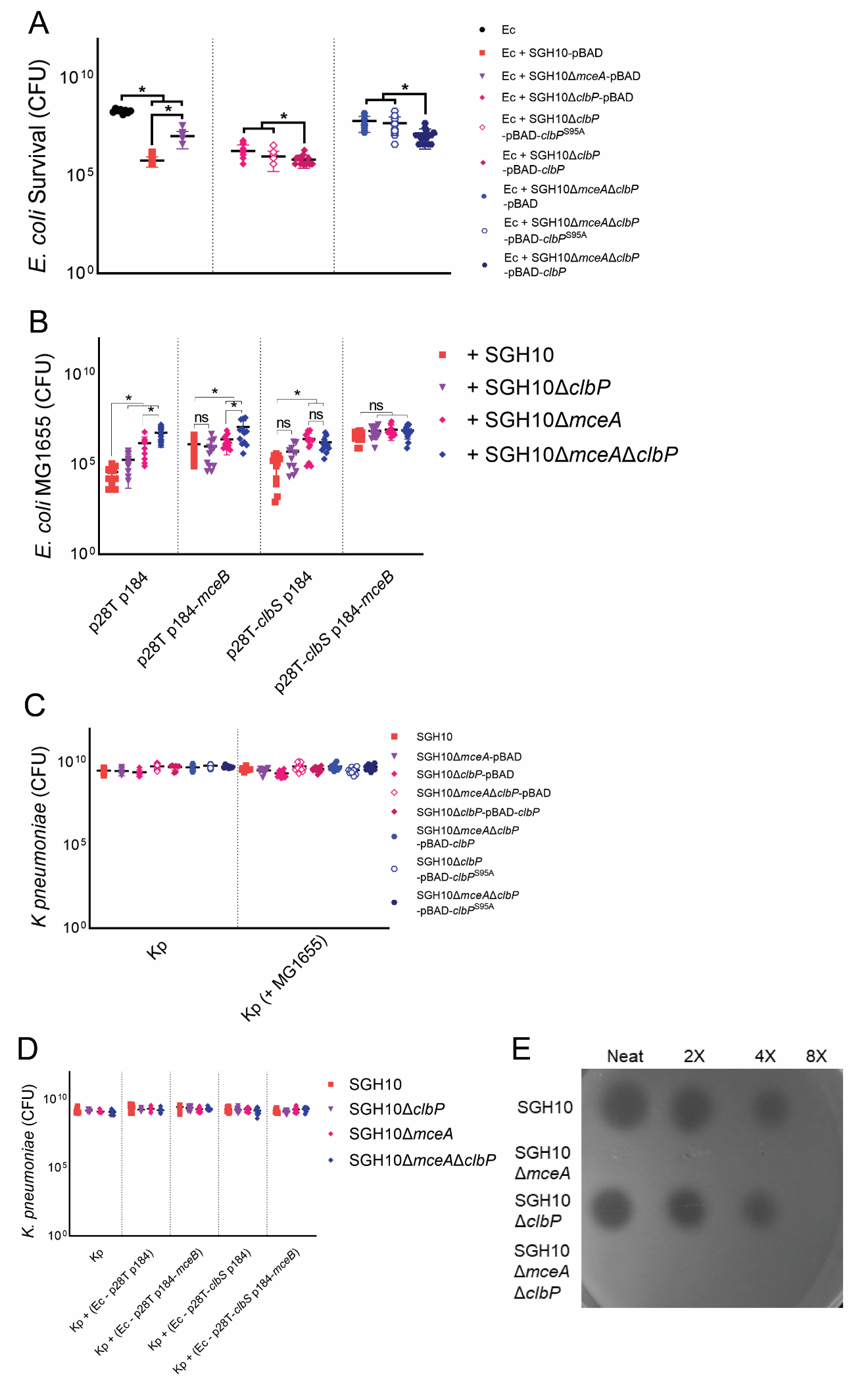


Supplementary Figure 6. Characterisation of SGH10Δ*clbP* and heterologous expression of the immunity proteins for mccE492 and colibactin in *E. coli*. A Complementation of SGH10Δ*clbP* restores killing of *E. coli* prey. SGH10, SGH10∆*mceA*, SGH10∆*clbP* and SGH10∆*mceA*∆*clbP* carrying either the pBAD control vector or pBAD expressing either wildtype ClbP or ClbP^S95A^ was competed with *E. coli* MG1655 for 24 h and *E. coli* survival was plotted. B MceB and ClbS were expressed in *E. coli* MG1655 to determine if they could protect against colibactin and mccE492 dependent killing. *E. coli* MG1655 carrying either p28T and p184, p28T and p184-*mceB*, p28T-*clbS* and p184, and p28T-*clbS* and p184-*mceB* was competed with SGH10 and mutants for 24 h and *E. coli* survival plotted. C The growth of complemented *K. pneumoniae* mutants co-cultured with *E. coli* MG1655 and D *K. pneumoniae* co-cultured with *E. coli* MG1655 with the p28T, p28T-*clbS*, p184 and p184-*mceB* vectors after 24h. E Semi-quantitative measurement of secreted mccE492. Sequential dilutions of culture supernatants from SGH10, SGH10Δ*mceA*, SGH10Δ*clbP* and SGH10Δ*mceA*Δ*clbP* were spotted on a soft agar containing *E. coli* MG1655 as prey. The plate was incubated at room temperature for 16 h before being imaged. In strains which secrete mccE492, clear halos indicate inhibition of growth in *E. coli*.


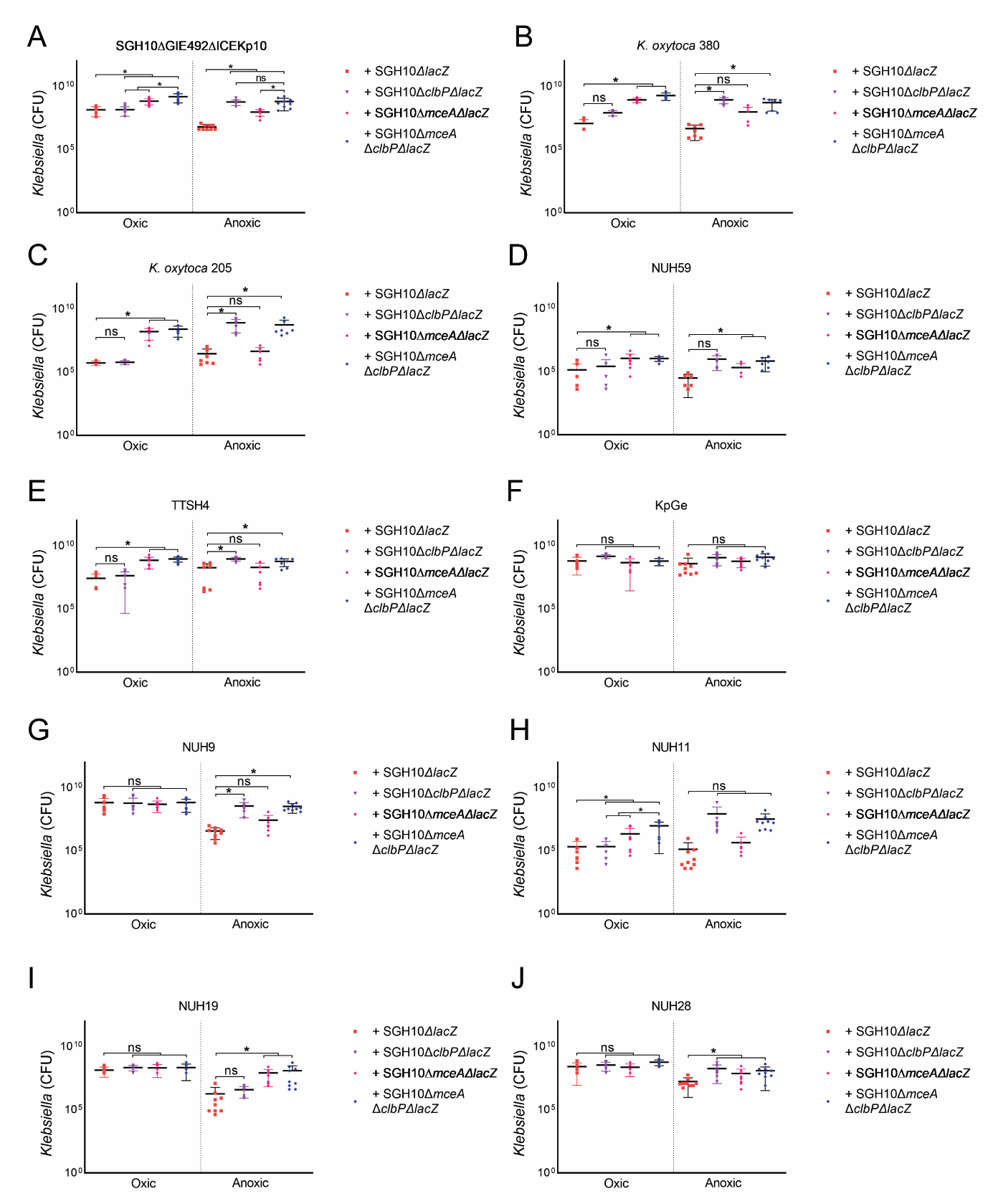


Supplementary Figure 7. Killing of *Klebsiella* prey. A SGH10ΔGIE492ΔICEKp10, B *K. oxytoca* 380, C *K. oxytoca* 205, D NUH59, E TTSH4, F KpGe, G NUH9, H NUH11, I NUH19 and J NUH28 were competed with SGH10 mutants on solid media under oxic and anoxic conditions for 24 h. *K. pneumoniae* prey strains were differentiated from SGH10 predator strains by blue-white screening. Mean ± SD (n=4) was plotted and Dunnett’s multiple comparisons test was performed to determine differences in means. ns indicates no significance and * indicates *p*<0.05.


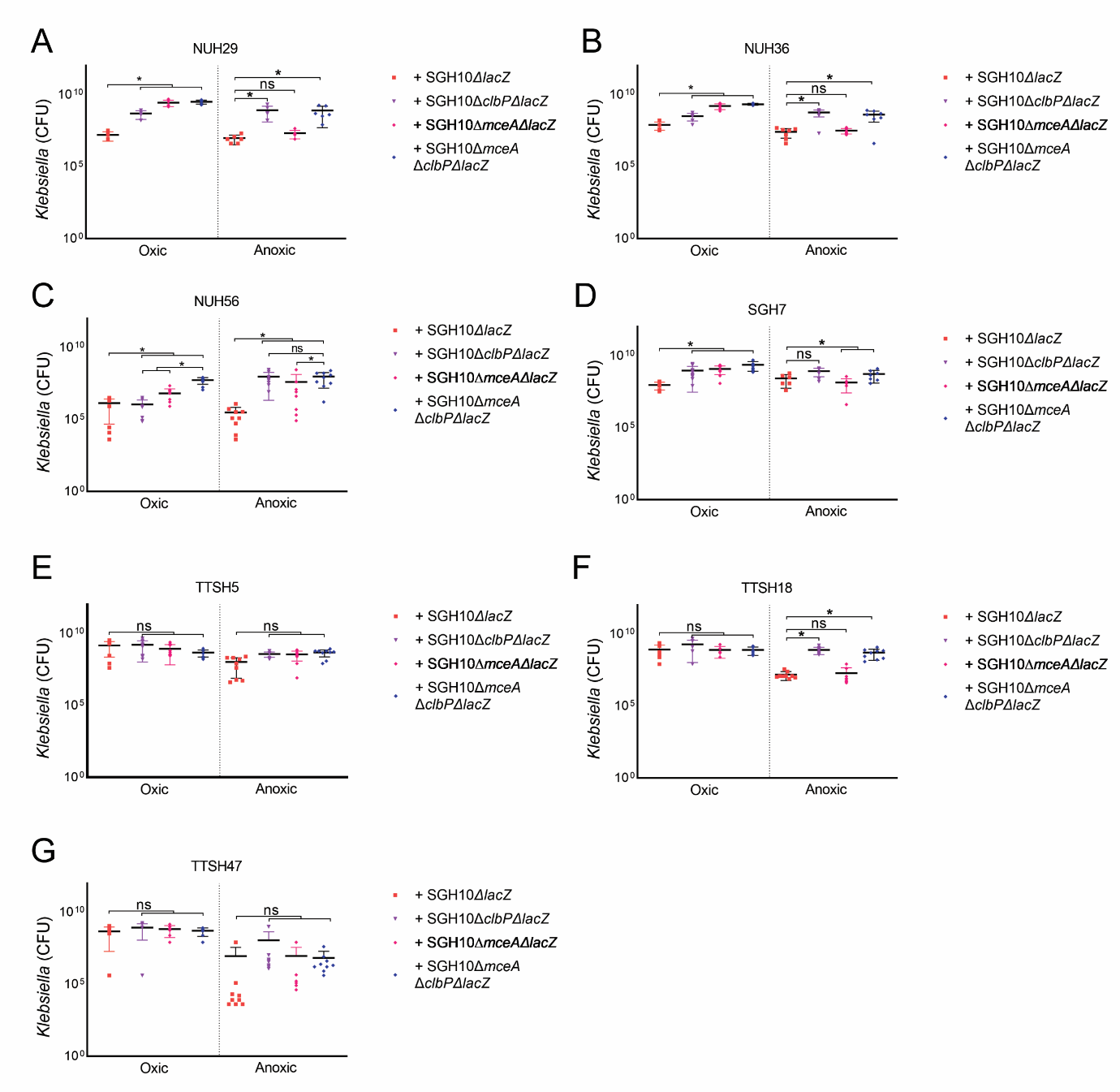


Supplementary Figure 8. Killing of *K. pneumoniae* prey isolates. A NUH29, B NUH36, C NUH56, DSGH7, E TTSH5, F TTSH18 and G TTSH47 were competed with SGH10 mutants on solid media under oxic and anoxic conditions for 24 h. *K. pneumoniae* prey strains were differentiated from SGH10 predator strains by blue-white screening. Mean ± SD (n=4) was plotted and Dunnett’s multiple comparisons test was performed to determine differences in means. ns indicates no significance and * indicates *p*<0.05.


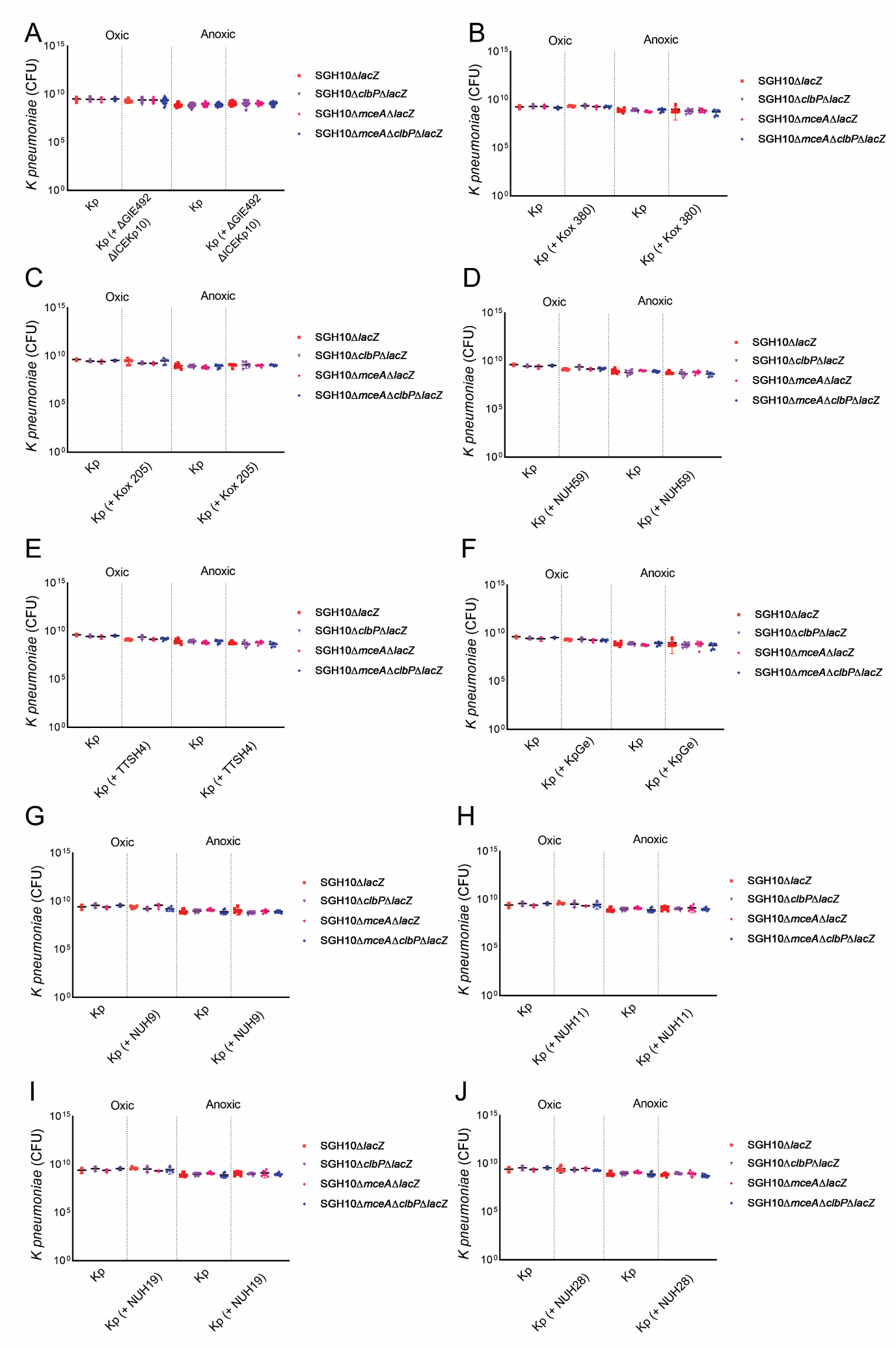


Supplementary Figure 9. Growth of *K. pneumoniae* during competition with *Klebsiella* prey. *K. pneumoniae* CFU after incubation with A SGH10ΔGIE492ΔICEKp10, B *K. oxytoca* 380, C *K. oxytoca* 205, D NUH59, E TTSH4, F KpGe, G NUH9, H NUH11, I NUH19 and J NUH28 prey on solid media under oxic and anoxic conditions for 24 h. Mean ± SD (n=4) was plotted and Dunnett’s multiple comparisons test was performed to determine differences in means. There were no significant differences in means between groups.


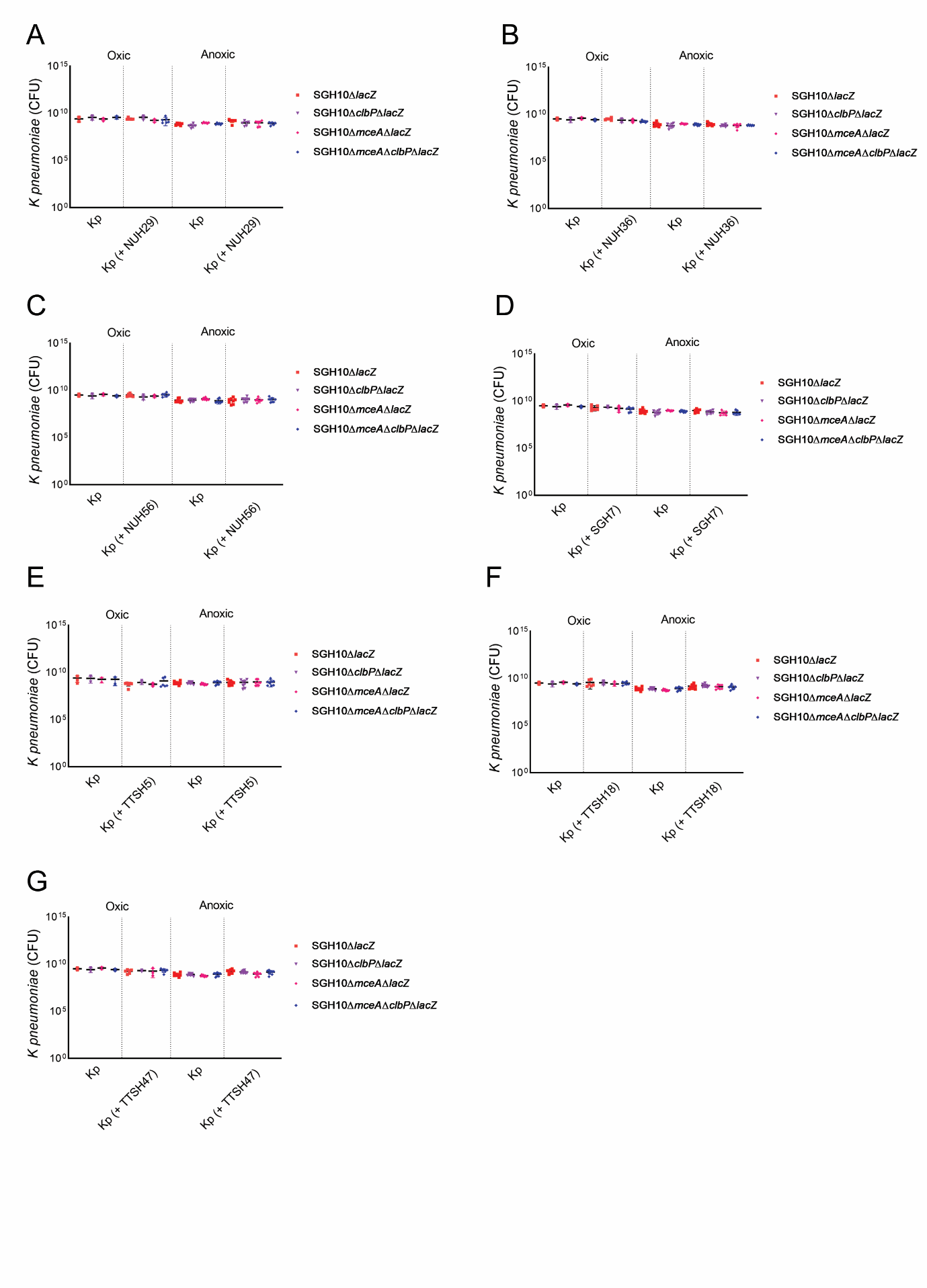


Supplementary Figure 10. Growth of *K. pneumoniae* during competition with *K. pneumoniae* isolates. *K. pneumoniae* strains were competed with A NUH29, B NUH36, C NUH56, D SGH7, E TTSH5, F TTSH18, and G TTSH47 prey on solid media under oxic and anoxic conditions for 24 h. Mean ± SD (n=4) was plotted and Dunnett’s multiple comparisons test was performed to determine differences in means. There were no significant differences in means between groups.


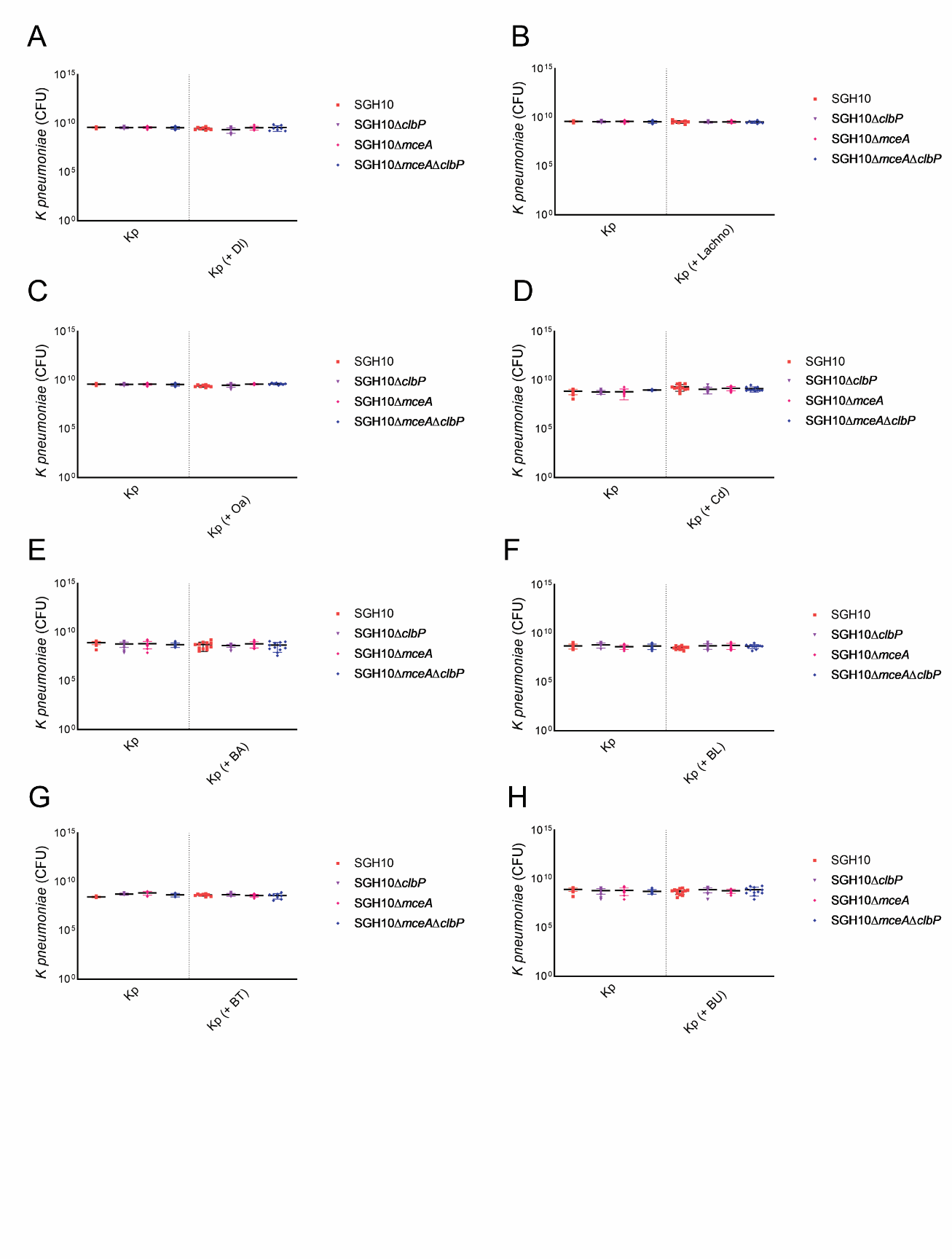


Supplementary Figure 11. Growth of *K. pneumoniae* during competition with obligate anaerobic bacteria prey. *K. pneumoniae* CFU after incubation with A *Dorea longicatena* (Dl) (BT), B *Lachnospiraceae* 24430 (Lachno), C *Oscillibacter acetigenes* (Oa), D Clostridioides difficile (Cd), E *Bifidobacterium adolescentis* (BA), F *Bifidobacterium longum* (BL), G *Bacteroides thetaiotaomicron* (BT) H *Bacteroides uniformis* (BU) prey at a ratio of 100:1 anaerobe to *K. pneumoniae* under anoxic conditions. The strains were competed together for 24h (BT) or 72 h (BU). Mean ± SD (n=4) was plotted and Dunnett’s multiple comparisons test was performed to determine differences in means. There were no significant differences in means between groups.


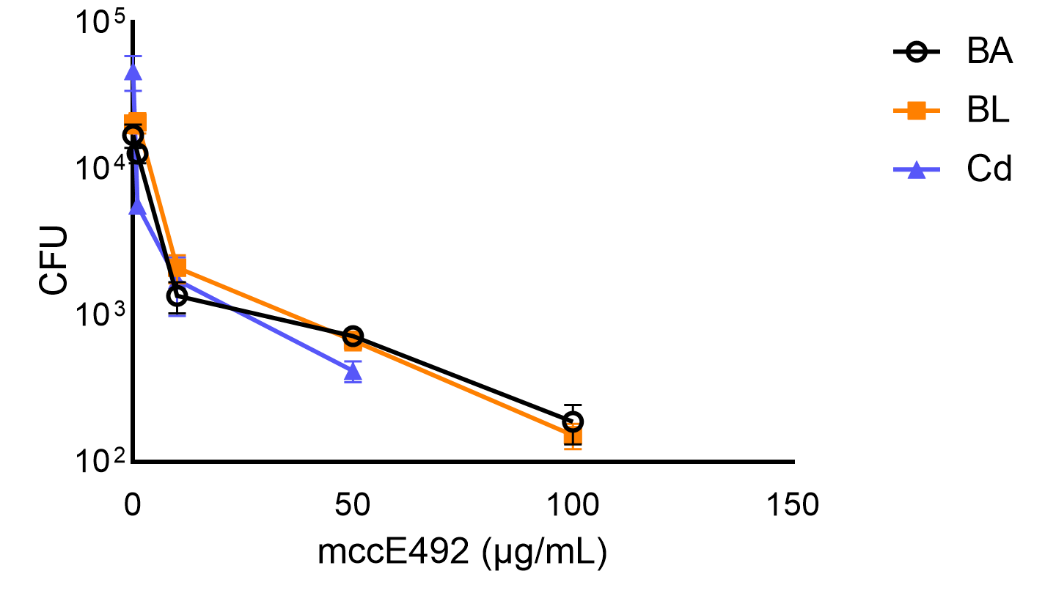


Supplementary Figure 12. Concentration-dependent killing of *C. difficile*, *B. adolescentis* and *B. longum* by mccE492. Bacterial CFU is plotted after a 6 h incubation with increasing concentrations of mccE492.


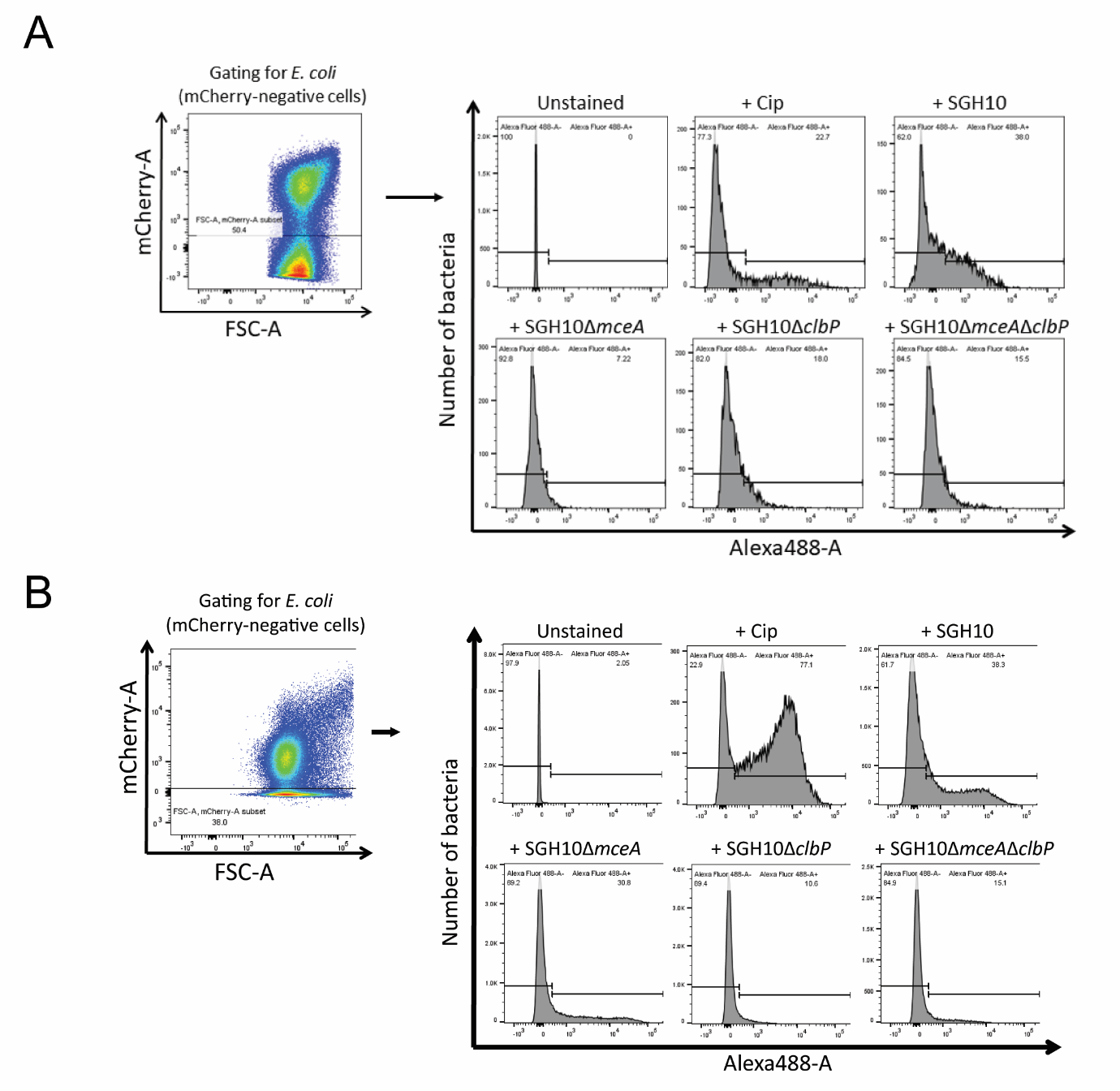


Supplementary Figure 13. Quantification of DNA damage in *E. coli* using the TUNEL assay. A *E. coli* MG1655 was co-cultured with HvKp SGH10 at an initial ratio of 10:1 for 4 h under oxic and B anoxic conditions. 50µg/mL ciprofloxacin (Cip) was used as a positive control for DNA damage. *K. pneumoniae* strains were tagged with an mApple reporter (mCherry) which allows us to distinguish between *K. pneumoniae* and *E. coli* populations by gating for mCherry-negative cells (*E. coli* prey). Representative flow plots of TUNEL staining (Alexa-488) in the *E. coli* prey are plotted.


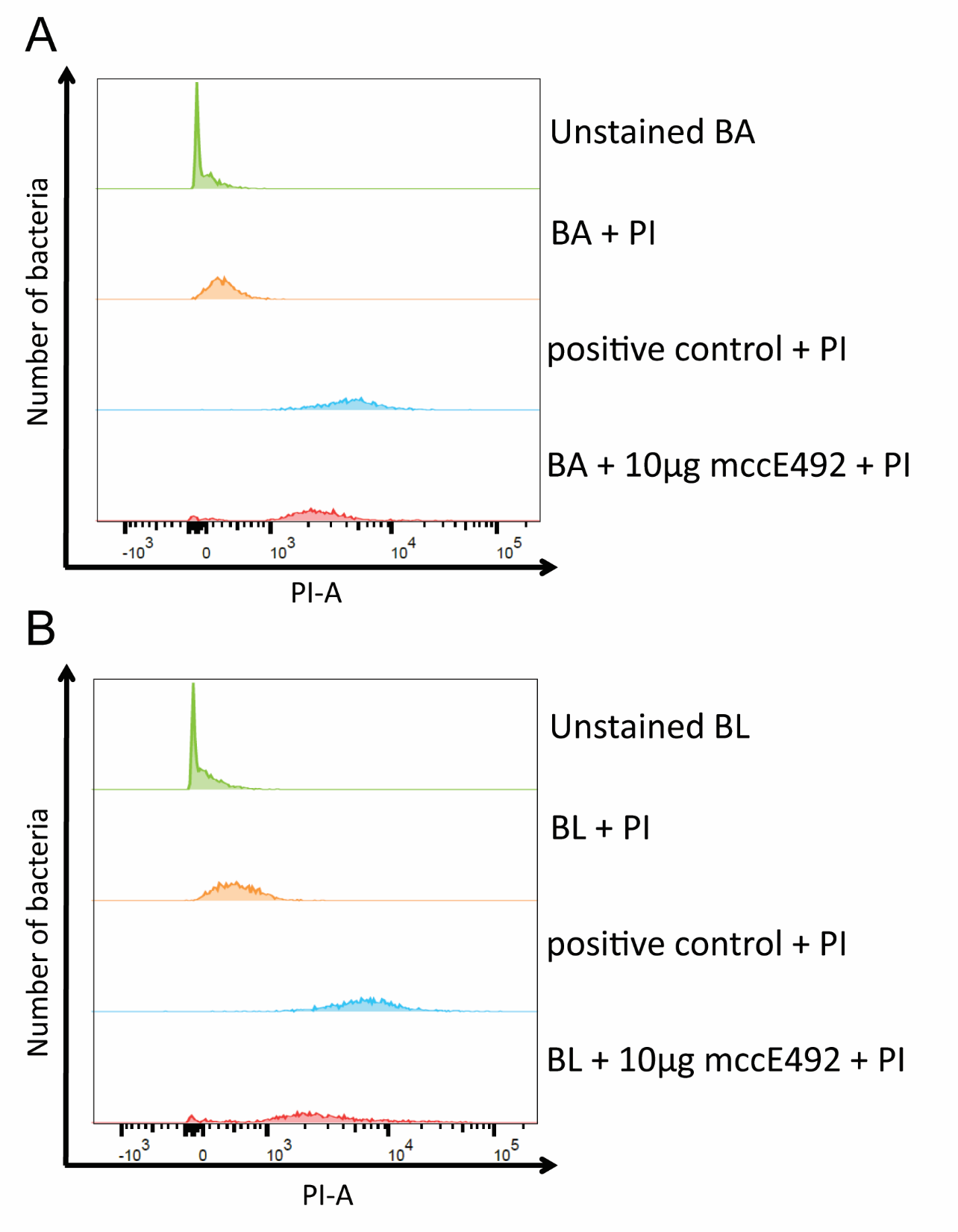


Supplementary Figure 14. Membrane perturbation of BA and BL by mccE492. A Representative flow plots of BA and B BL treated with mccE492 for 30 min before being stained with propidium iodide (PI). Positive controls were generated by treating BA and BL with lysozyme, mutanolysin and isopropanol.


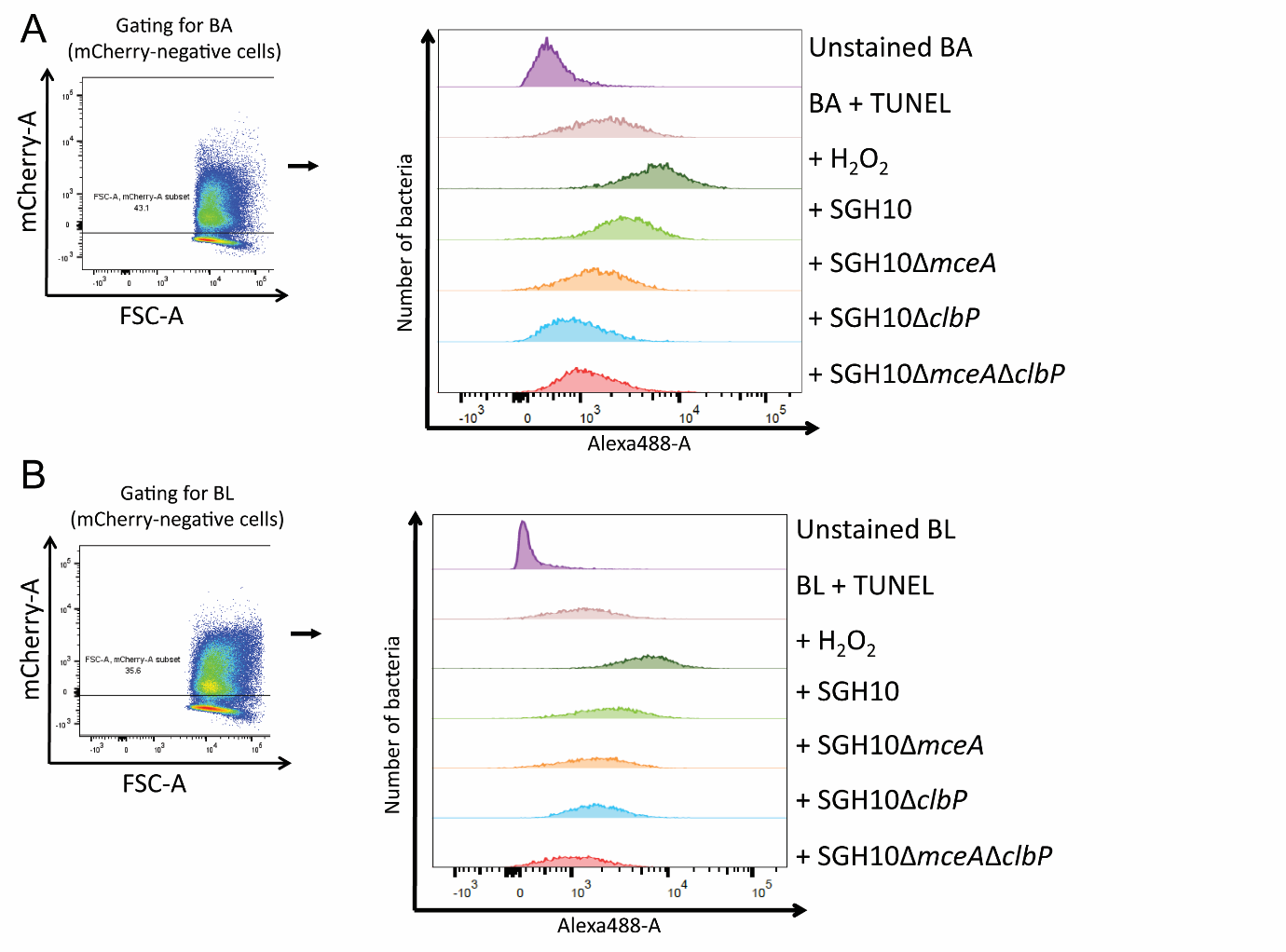


Supplementary Figure 15. Quantification of DNA damage in *B. adolescentis* (BA) and *B. longum* (BL) using the TUNEL assay. A BA and B BL were co-cultured with SGH10 at an initial ratio of 10:1 for 4 h under anoxic conditions. 1% H_2_O_2_ was used as a positive control for DNA damage. *K. pneumoniae* strains were tagged with an mApple reporter (mCherry) which allows us to distinguish between *K. pneumoniae* and prey populations by gating mCherry-negative cells (BA or BL). Representative flow plots of TUNEL staining (Alexa-488) in BA and BL are plotted.


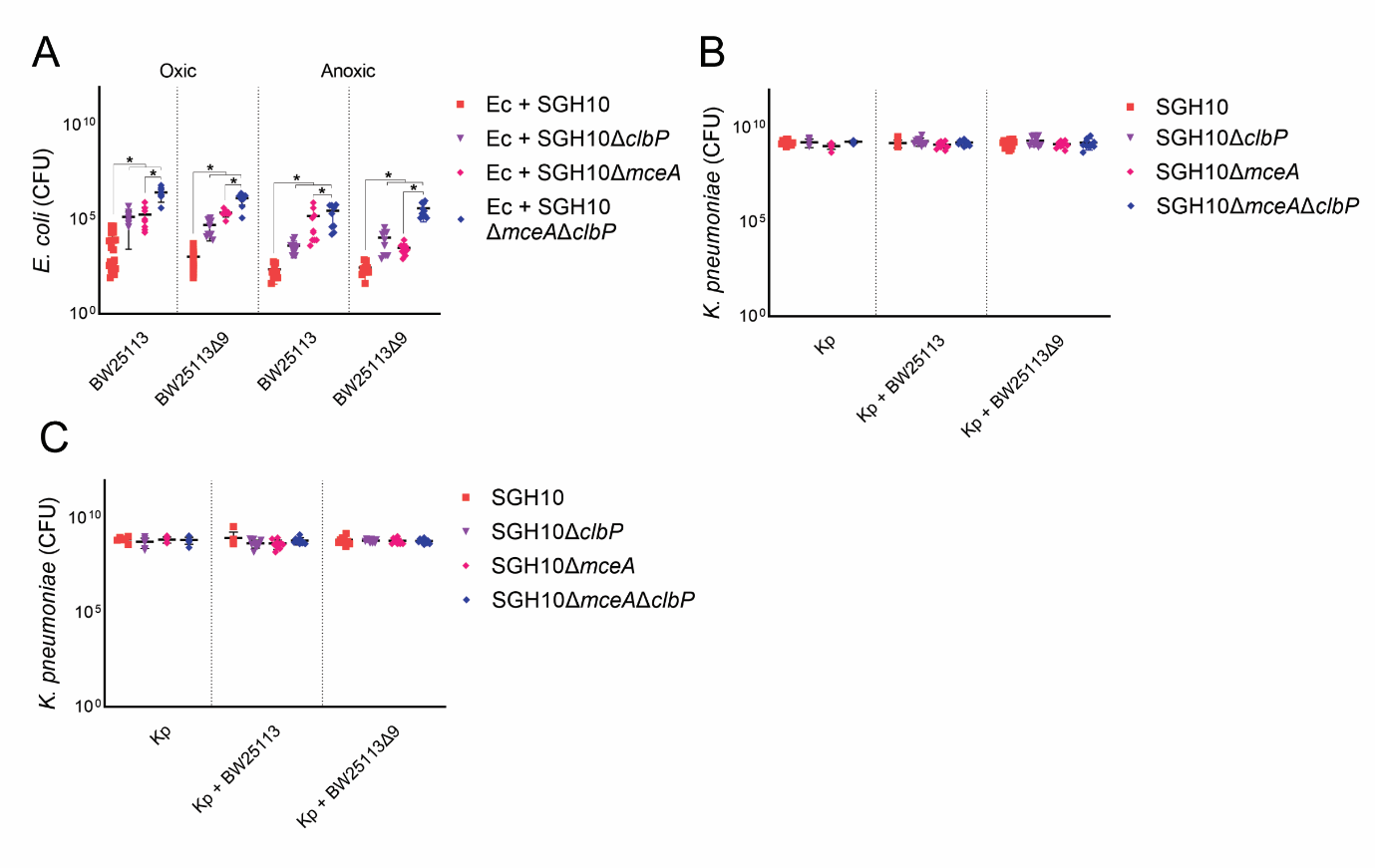


Supplementary Figure 16. To determine if prophages were involved in colibactin dependent killing of *E. coli*, *E. coli* BW25113 and Δ9 were competed with SGH10 and mutants for 24 h. A *E. coli*, and *K. pneumoniae* CFU under B oxic and C anoxic conditions is plotted. Mean ± SD (n=4) was plotted and Dunnett’s multiple comparisons test was performed to determine differences in means. * denotes *p*<0.05 and ** denotes *p*<0.01.

References

1. Molton, J. S. *et al.* Oral vs intravenous antibiotics for patients with *Klebsiella pneumoniae* liver abscess: A randomized, controlled noninferiority study. *Clinical Infectious Diseases* **71**, 952–959 (2020).

2. Wyres, K. L. *et al.* Genomic surveillance for hypervirulence and multi-drug resistance in invasive *Klebsiella pneumoniae* from South and Southeast Asia. *Genome Med* **12**, 11 (2020).

3. Baker, K. S. *et al.* The Murray collection of pre-antibiotic era Enterobacteriacae: a unique research resource. *Genome Med* **7**, 97 (2015).

4. Lam, M. M. C. *et al.* Population genomics of hypervirulent *Klebsiella pneumoniae* clonal-group 23 reveals early emergence and rapid global dissemination. *Nat Commun* **9**, 2703 (2018).

5. Lam, M. M. C. *et al.* A genomic surveillance framework and genotyping tool for *Klebsiella pneumoniae* and its related species complex. *Nat Commun* **12**, 4188 (2021).

6. Hennart, M. *et al.* A dual barcoding approach to bacterial strain nomenclature: genomic taxonomy of *Klebsiella pneumoniae* strains. *Molecular Biology and Evolution* **39**, msac135 (2022).

7. Katoh, K. & Standley, D. M. MAFFT Multiple sequence alignment software version 7: Improvements in performance and usability. *Molecular Biology and Evolution* **30**, 772–780 (2013).

8. Minh, B. Q. *et al.* IQ-TREE 2: New models and efficient methods for phylogenetic Inference in the genomic era. *Molecular Biology and Evolution* **37**, 1530–1534 (2020).

9. Kalyaanamoorthy, S., Minh, B. Q., Wong, T. K. F., Von Haeseler, A. & Jermiin, L. S. ModelFinder: fast model selection for accurate phylogenetic estimates. *Nat Methods* **14**, 587–589 (2017).

10. Hoang, D. T., Chernomor, O., Von Haeseler, A., Minh, B. Q. & Vinh, L. S. UFBoot2: Improving the Ultrafast Bootstrap Approximation. *Molecular Biology and Evolution* **35**, 518–522 (2018).

11. Marcoleta, Berríos-Pastén, C., Nuñez, G., Monasterio, O. & Lagos, R. *Klebsiella pneumoniae* asparagine tDNAs are integration hotspots for different genomic islands encoding microcin E492 production determinants and other putative virulence factors present in hypervirulent strains. *Front. Microbiol.* **7**, (2016).

12. Altschul, S. F., Gish, W., Miller, W., Myers, E. W. & Lipman, D. J. Basic local alignment search tool. *Journal of Molecular Biology* **215**, 403–410 (1990).

13. Wick, R. R., Judd, L. M. & Holt, K. E. Assembling the perfect bacterial genome using Oxford Nanopore and Illumina sequencing. *PLoS Comput Biol* **19**, e1010905 (2023).

14. Parks, D. H., Imelfort, M., Skennerton, C. T., Hugenholtz, P. & Tyson, G. W. CheckM: assessing the quality of microbial genomes recovered from isolates, single cells, and metagenomes. *Genome Res.* **25**, 1043–1055 (2015).

15. Sullivan, M. J., Petty, N. K. & Beatson, S. A. Easyfig: a genome comparison visualizer. *Bioinformatics* **27**, 1009–1010 (2011).

16. Tan, Y. H., Chen, Y., Chu, W. H. W., Sham, L. & Gan, Y. Cell envelope defects of different capsule‐null mutants in K1 hypervirulent *Klebsiella pneumoniae* can affect bacterial pathogenesis. *Mol Microbiol* **113**, 889–905 (2020).

17. Chen, S., Zhou, Y., Chen, Y. & Gu, J. fastp: an ultra-fast all-in-one FASTQ preprocessor. *Bioinformatics* **34**, i884–i890 (2018).

18. Li, H. Aligning sequence reads, clone sequences and assembly contigs with BWA-MEM. Preprint at http://arxiv.org/abs/1303.3997 (2013).

19. Li, H. *et al.* The sequence alignment/map format and SAMtools. *Bioinformatics* **25**, 2078–2079 (2009).

20. Wood, D. E., Lu, J. & Langmead, B. Improved metagenomic analysis with Kraken 2. *Genome Biol* **20**, 257 (2019).

21. Beresford-Jones, B. S. *et al.* The mouse gastrointestinal bacteria catalogue enables translation between the mouse and human gut microbiotas via functional mapping. *Cell Host & Microbe* **30**, 124-138.e8 (2022).

22. Lu, J., Breitwieser, F. P., Thielen, P. & Salzberg, S. L. Bracken: estimating species abundance in metagenomics data. *PeerJ Computer Science* **3**, e104 (2017).

23. Dixon, P. VEGAN, a package of R functions for community ecology. *Journal of Vegetation Science* **14**, 927–930 (2003).

24. Wickham, Hadley. *ggplot2 Elegant graphics for data analysis*. (Springer-Verlag, New York, 2016).

25. Mallick, H. *et al.* Multivariable association discovery in population-scale meta-omics studies. *PLoS Comput Biol* **17**, e1009442 (2021).

26. Marcoleta *et al.* Microcin E492 amyloid formation Is retarded by posttranslational modification. *Journal of Bacteriology* **195**, 3995–4004 (2013).

27. Lagos, R. *et al.* Structure, organization and characterization of the gene cluster involved in the production of microcin E492, a channel-forming bacteriocin. *Molecular Microbiology* **42**, 229–243 (2001).
